# A Regulatory Network for Coordinated Flower Maturation

**DOI:** 10.1371/journal.pgen.1002506

**Published:** 2012-02-09

**Authors:** Paul H. Reeves, Christine M. Ellis, Sara E. Ploense, Miin-Feng Wu, Vandana Yadav, Dorothea Tholl, Aurore Chételat, Ina Haupt, Brian J. Kennerley, Charles Hodgens, Edward E. Farmer, Punita Nagpal, Jason W. Reed

**Affiliations:** 1 Department of Biology, University of North Carolina at Chapel Hill, Chapel Hill, North Carolina, United States of America; 2 Department of Biological Sciences, Virginia Tech University, Blacksburg, Virginia, United States of America; 3 Department of Plant Molecular Biology, Biophore, University of Lausanne, Lausanne, Switzerland; 4 Max-Planck Institute for Chemical Ecology, Jena, Germany; 5 College of Science, King Saud University, Riyadh, Saudi Arabia; University of California San Diego, United States of America

## Abstract

For self-pollinating plants to reproduce, male and female organ development must be coordinated as flowers mature. The Arabidopsis transcription factors AUXIN RESPONSE FACTOR 6 (ARF6) and ARF8 regulate this complex process by promoting petal expansion, stamen filament elongation, anther dehiscence, and gynoecium maturation, thereby ensuring that pollen released from the anthers is deposited on the stigma of a receptive gynoecium. ARF6 and ARF8 induce jasmonate production, which in turn triggers expression of *MYB21* and *MYB24*, encoding R2R3 MYB transcription factors that promote petal and stamen growth. To understand the dynamics of this flower maturation regulatory network, we have characterized morphological, chemical, and global gene expression phenotypes of *arf*, *myb*, and jasmonate pathway mutant flowers. We found that MYB21 and MYB24 promoted not only petal and stamen development but also gynoecium growth. As well as regulating reproductive competence, both the ARF and MYB factors promoted nectary development or function and volatile sesquiterpene production, which may attract insect pollinators and/or repel pathogens. Mutants lacking jasmonate synthesis or response had decreased *MYB21* expression and stamen and petal growth at the stage when flowers normally open, but had increased *MYB21* expression in petals of older flowers, resulting in renewed and persistent petal expansion at later stages. Both auxin response and jasmonate synthesis promoted positive feedbacks that may ensure rapid petal and stamen growth as flowers open. MYB21 also fed back negatively on expression of jasmonate biosynthesis pathway genes to decrease flower jasmonate level, which correlated with termination of growth after flowers have opened. These dynamic feedbacks may promote timely, coordinated, and transient growth of flower organs.

## Author Summary

Perfect flowers have both male organs that produce and release pollen and female organs that make and harbor seeds. Flowers also often attract pollinators using visual or chemical signals. So that male, female, and pollinator attraction functions occur at the right time, flower organs must grow and mature in a coordinated fashion. In the model self-pollinating plant Arabidopsis, a transcriptional network regulates genes that ensure coordinated growth of different flower organs, as well as pollen release and gynoecium (female) competence to support pollination. This network also regulates nectary development and production of volatile chemicals that may attract or repel insects. We have studied growth, chemical signal levels, and gene expression in mutants affected in components of this network, in order to determine how flower growth is controlled. Several plant hormones act in a cascade that promotes flower maturation. Moreover, regulatory feedback loops affect the timing and extent of developmental steps. Positive feedbacks may ensure that the development of different flower organs is coordinated and rapid, whereas negative feedbacks may allow growth to cease once flowers have opened. Our results provide a framework to understand how flower opening and reproduction are coordinated in Arabidopsis and other flowering plants.

## Introduction

In typical angiosperms, late in flower development, sepals open to expose the inner organs; the petals, stamen filaments, and style elongate; the anthers dehisce to release pollen; and the stigma and transmitting tract mature so as to permit pollen germination and pollen tube growth. These events often occur quite quickly, and are transient, so that flowers open and pollinate, but then stop growing. Effective reproduction therefore requires accurate coordination of these events. Variation in spatial arrangement and timing of maturation of different organs may affect the pollination mode and the mating system. In plants with self-pollinating flowers such as *Arabidopsis thaliana*, stamens and gynoecium grow to about the same length and mature synchronously, allowing efficient self-fertilization [1]. In outcrossing plants, differential growth of stamens and style or staggered timing of anther and gynoecium maturation can instead promote cross-pollination.

The Arabidopsis transcription factors AUXIN RESPONSE FACTOR 6 (ARF6/*At1g30330*) and ARF8/*At5g37020* act partially redundantly to promote late stages in petal, stamen and gynoecium development. *arf6 arf8* double null mutant flowers arrest at flower stage 12 as closed buds with short petals, short stamen filaments, undehisced anthers, and immature gynoecia with short stigmatic papillae and poor support of pollen tube growth, and are largely male- and female-sterile [2]–[4]. *arf6* and *arf8* single mutants and sesquimutants (homozygous for one mutation and heterozygous for the other) have delayed stamen filament elongation and decreased fecundity. *ARF6* and *ARF8* are each expressed in multiple flower tissues including sepals, petals, stamen filaments, style, transmitting tract, ovule funiculi, and nectaries [2], [4]. ARF6 and ARF8 thus act in several organs to promote the transition from closed buds to mature fertile flowers, and to ensure coordinated development of male and female organs, leading to efficient self-fertilization.

*arf6-2 arf8-3* flowers have very low jasmonic acid (JA) levels and decreased expression of several jasmonate biosynthesis genes, and exogenous methyl jasmonate (MeJA) rescued the petal elongation and anther dehiscence defects, but not the stamen elongation defect or gynoecium arrest, of *arf6 arf8* flowers [2], [3]. Mutants affected in jasmonate synthesis or signaling similarly have delayed stamen growth and indehiscent anthers [5]–[8]. Similarly to stamens, petals of jasmonate pathway mutants have been reported to have delayed growth [6]. However, in contrast, other groups have reported that petals of jasmonate pathway mutants are larger than those of wild-type flowers [5], [8], [9]. Jasmonates can inhibit petal expansion by activating alternative splicing of a *bHLH31/BPE/At1g59640* transcript [9], [10]. *arf8* mutants also had enlarged petals, suggesting that ARF8 and BPE act in a common pathway [11]. These results indicate that ARF6 and ARF8 trigger anther dehiscence by promoting jasmonate production, can promote or inhibit petal growth through jasmonate-dependent pathways, and regulate other aspects of flower maturation independently of jasmonate.

The role of jasmonate in stamen development was investigated in more detail by examining MeJA-induced global gene expression changes in the stamens of jasmonate-deficient *opr3* mutant plants [8], [12]. Two closely related R2R3 MYB transcription factor genes, *MYB21/At3g27810* and *MYB24/At5g40350* [13], were rapidly induced by jasmonate. *myb21* mutants had short stamen filaments and petals, and *myb21 myb24* double mutants had indehiscent anthers. These phenotypes were not rescued by exogenous JA or MeJA application, indicating that MYB21 and MYB24 act downstream of jasmonate signaling to promote stamen and petal development [12], [14]. Gibberellin-deficient mutants also have delayed stamen development, decreased JA level, and decreased expression of *MYB21*, *MYB24*, and a third closely related gene, *MYB57/At3g01530* [14]. A fourth closely related gene, *MYB108/At3g06490*, also contributes to stamen development partially redundantly with *MYB24* [15]. *MYB57* and *MYB108* are also induced by jasmonate. *MYB108* has also been isolated as *BOTRYTIS OVERSENSITIVE 1* (*BOS1*), and is required for JA-mediated biotic and abiotic stress responses [16]. MYB21 and MYB24 can activate transcription, and overexpression of *MYB21* or *MYB24* caused defects in flower development [17]–[20]. Other genes encoding members of this clade, *MYB2*, *MYB62*, *MYB78*, *MYB112* and *MYB116*, were not appreciably expressed in flowers [21].

To understand how these components interact to regulate flower maturation, we have analyzed the relative timing of flower organ growth in *arf*, *myb*, and jasmonate pathway mutants, and compared expression of *MYB* and jasmonate pathway genes in wild-type and mutant flowers. These analyses suggest a hierarchical regulatory pathway that triggers flower maturation, and also reveal contrasting effects of jasmonate on petal growth at different developmental stages. Analyses of global gene expression patterns in wild-type, *myb21 myb24*, and *arf6 arf8* flowers reveal that the flower maturation network controls putative chemical attractant functions of flowers, and that both positive and negative feedback loops control auxin and jasmonate responses during flower maturation.

## Results

### *MYB* Genes Are Expressed Downstream of ARF6 and ARF8 in Multiple Flower Organs

**Figure 1:**
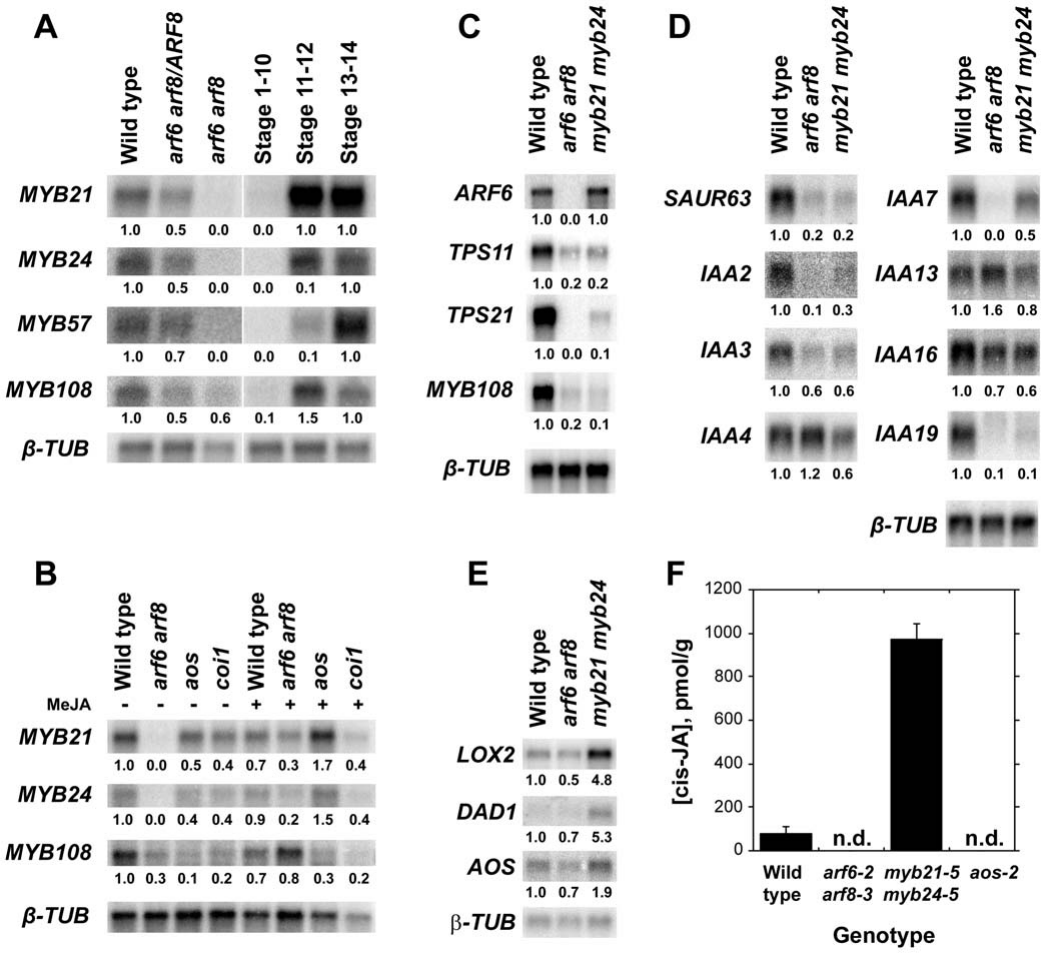
Gene expression and jasmonate production in wild-type and mutant flowers. (A–B) RNA gel blot hybridization using *MYB21*, *MYB24*, *MYB57* and *MYB108* probes. (A) RNA from wild-type, *arf6-2 arf8-3/ARF8* and *arf6-2 arf8-3* inflorescences (left panel), and wild-type stage 1–10, stage 11–12 and stage 13–14 flowers (right panel). (B) RNA from untreated (left panel) or MeJA treated (right panel) wild-type, *arf6-2 arf8-3*, *aos-2* and *coi1-1* inflorescences. (C) RNA gel blot hybridization using *ARF6*, *TPS11*, *TPS21*, *MYB108* and *SAUR63* probes. RNA from wild-type, *arf6-2 arf8-3* and *myb21-5 myb24-5* inflorescences. (D) RNA gel blot hybridization using *SAUR63*, *IAA2*, *IAA3*, *IAA4*, *IAA7*, *IAA13*, *IAA16* and *IAA19* probes. RNA from wild-type, *arf6-2 arf8-3* and *myb21-5 myb24-5* stage 12-13 flowers. (E) RNA gel blot hybridization using *LOX2*, *DAD1*, and *AOS* probes. PolyA^+^ RNA from wild-type, *arf6-2 arf8-3* and *myb21-5 myb24-5* stage 12–13 flowers. In A–E, numbers beneath each band indicate measured signal level relative to the *β-TUBULIN* control. (F) cis-JA concentrations in wild-type, *arf6-2 arf8-3*, *myb21-5 myb24-5*, and *aos-2* stage 12-13 flowers. Data are means of two measurements ± SD. n.d., not detected.

**Figure 2:**
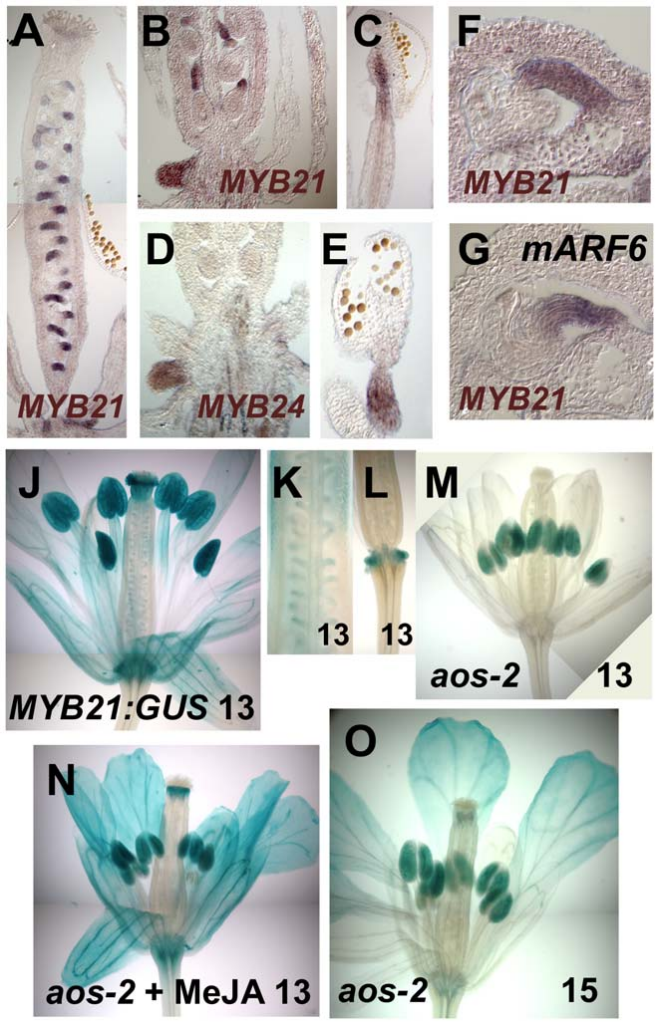
Expression patterns of *MYB21* and *MYB24*. (A–C) *In situ* hybridization with a *MYB21* antisense probe in stage 12 wild-type gynoecia (A,B) or stamen filament (C). (D,E) *In situ* hybridization with a *MYB24* antisense probe in stage 12 wild-type nectary (D) and stament filament (E). (F) *MYB21 in situ* hybridization in a wild-type ovule. (G) *MYB21 in situ* hybridization in a *mARF6* ovule. (J–O) X-Gluc staining of *P_MYB21_:MYB21:GUS* flowers. (J) Stage 13 wild-type whole flower. (K) Gynoecium showing ovule funiculi. (L) Gynoecium base showing nectary. (M–O) *aos-2 P_MYB21_:MYB21:GUS* flowers at stage 13 (M), (N) MeJA-treated stage 13, (O) Stage 15 untreated.

Before characterizing mutant phenotypes, we examined expression of *MYB* genes in wild-type, *arf6-2 arf8-3*, and jasmonate pathway mutant flowers. In wild-type flowers, *MYB21* and *MYB24* were first expressed at stages 11–12 shortly before flower opening, whereas in *arf6-2 arf8-3* flowers *MYB21* and *MYB24* mRNAs were almost undetectable (Figure 1A; Figure S1A, S1C) [2]. Conversely, *ARF6* and *ARF8* mRNA levels were normal in *myb21-5 myb24-5* flowers (Figure 1C, Table S3). *MYB21* and *MYB24* were also underexpressed in jasmonate-deficient *aos-2* mutant inflorescence apices and in jasmonate-resistant *coi1-1* apices (Figure 1B). Both *MYB21* and *MYB24* baseline expression levels were lower in *arf6-2 arf8-3* inflorescences than in *aos-2* or *coi1-1* inflorescences (Figure 1B). Exogenous methyl jasmonate induced *MYB21* and *MYB24* genes in *arf6-2 arf8-3* and *aos-2* mutant inflorescence apices, but not in *coi1-1* apices (Figure 1B) [12], [14], [15]. *P_35S_:ARF6* plants that overexpress *ARF6* did not have increased *MYB21* mRNA level (Figure S1A); and *P_ARF6_:mARF6* plants expressing an *ARF6* transgene that is immune to regulation by *miR167*, and which have an expanded *ARF6* expression domain in the ovules [4], did not have a similarly expanded *MYB21* expression domain (Figure 2F, 2G). These results suggest that ARF6 and ARF8 induce these *MYB* genes indirectly, at least partly by increasing jasmonate levels. Exogenous MeJA only partially restored *MYB21* and *MYB24* expression and stamen and petal growth in *arf6-2 arf8-3* flowers (Figure 1B) [2], raising the possibility that ARF6 and ARF8 may also regulate *MYB21* and *MYB24* by additional jasmonate-independent mechanisms.

By *in situ* hybridization and using transgenic plants carrying a *P_MYB21_:MYB21:GUS* protein fusion reporter, we detected *MYB21* expression in sepals, petals, the apical part of stamen filaments, the style, ovule funiculi, and nectaries of stage 13 and 14 flowers (Figure 2A–2C, 2F, 2J–2L; Figure S1D). In the *aos-2* jasmonate-deficient background, *P_MYB21_:MYB21:GUS* expression was decreased in these organs, but was restored by exogenous methyl jasmonate (Figure 2M, 2N). *MYB24* and *P_MYB24_:MYB24:GUS* were likewise expressed in stamen filaments, style, and nectaries of stage 13 and 14 flowers, but not in ovule funiculi (Figure 2D, 2E; Figure S1E). Available microarray expression data are consistent with expression of both *MYB21* and *MYB24* in sepals, petals, stamens and carpels [21]. Expression of *P_MYB21_:MYB21:GUS* and *P_MYB24_:MYB24:GUS* in anthers or pollen (Figure 2, Figure S1) is likely an artifact of our fusion constructs, because *in situ* hybridizations revealed stamen filament but not anther expression (Figure 2C, 2E) [14]; microarray data from dry or germinated pollen revealed no expression of *MYB21* or *MYB24* [22]; and X-Gluc staining was present in anthers of *arf6-2 arf8-3 P_MYB21_:MYB21:GUS* plants although *arf6-2 arf8-3* flowers lacked detectable *MYB21* transcript (Figure 1A, 1B; Figure S1A–S1C).

### MYB21 and MYB24 Promote Petal and Stamen Development

**Figure 3:**
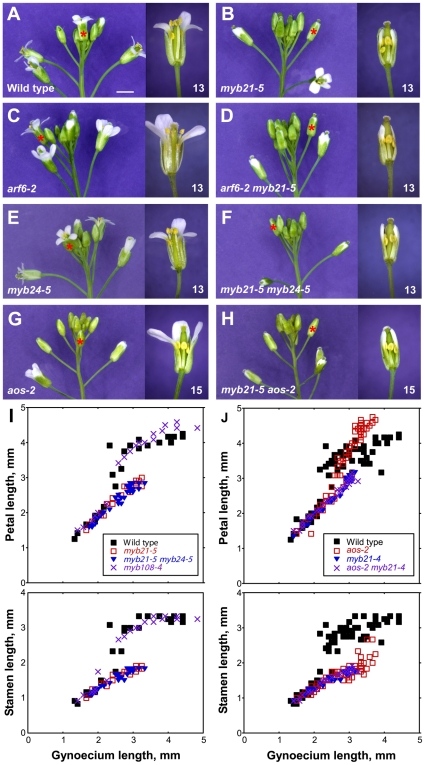
Inflorescence apices and flower phenotypes of *myb21*, *myb24*, *myb108*, and *aos* mutants. (A–H) Photographs of inflorescences (left panels) and individual flowers (right panels) of indicated genotypes. Asterisks indicate the position of the first open flower (stage 13) in the inflorescences shown, or the corresponding flower based upon bud size and position compared to a wild-type inflorescence. Individual flowers shown in the right panels are the first open flower (stage 13, A–F) or the fourth open flower (stage 15, G–H). Some sepals and petals have been removed to show inner organs. Scale bar: left panels, 3 mm, right panels, 1 mm. (I, J) Scatter plots showing petal and stamen lengths relative to gynoecium length of individual flowers of indicated genotypes. In I, data from a single experiment are shown. In J, measurements from two experiments were combined. Figure S2 shows similar data for additional genotypes.

To determine timing of stamen and petal growth in flowers of different genotypes, we measured organ lengths of flowers along a developmental series from closed buds to open flowers (Figure 3) [23]. Wild-type gynoecia elongated at a fairly constant rate through these stages, so that gynoecium length provided an internal reference for developmental stage. In addition, in independent experiments we measured organ lengths of flowers at defined positions on the inflorescence relative to the position of the first open flower in wild-type plants (Table S1). In wild-type Arabidopsis flowers, sepals stopped growing at stage 12, shortly before flowers opened [1], [23]. Petals and stamens grew slowly through early stages, but grew much more rapidly at stage 12 and stage 13, when the flowers opened (Figure 3A, 3I, 3J). Wild-type flowers generally self-pollinated as they opened. Just after this stage, petals and stamens stopped elongating, and about two days thereafter they began to senesce [1], [24].

*arf6-2* and *arf8-3* single mutants had delayed petal and stamen growth compared to wild type, but at a slightly later stage *arf6-2* and *arf8-3* mutant petals and stamens did reach wild-type lengths relative to gynoecium length (Figure 3C, Figure S2A). Although *arf8-3* mutants have been reported to have longer and wider petals than wild type [11], under our growth conditions petals of *arf6-2* and *arf8-3* flowers appeared wider but were not longer than wild-type petals. *arf6-2 arf8-3* double mutant flowers arrested with short stamens, petals, and gynoecia (Figure S2A) [2], [3].

We recovered the presumed null mutations *myb21-4* and *myb21-5*, each of which has a stop codon in the *MYB21* coding sequence, in a screen for *arf6-2* enhancers (Figure S3); and we used available T-DNA insertion alleles in *MYB24* (Materials and Methods, Figure S4, Table S2). *arf6-2 myb21-4* and *arf6-2 myb21-5* plants had flower buds with small unreflexed petals and short stamens, and set seed only when manually pollinated (Figure 3D, Table S1). The *myb21-5* mutation also enhanced *arf8-3* phenotypes, but did not affect organ lengths of the more severely affected *arf6-2 arf8-3* flowers (Table S1), indicating that *MYB21* can be placed in the same genetic pathway as *ARF6* and *ARF8*.

Similarly to other *myb21* mutants [12], [14], [20], *myb21-4* and *myb21-5* single mutant flowers had short petals, short stamens with reduced epidermal cell length, and delayed flower opening and anther dehiscence (Figure 3B, 3I, Figure S2C, Figure S5A–S5C, Table S1). The *myb21-4* and *myb21-5* mutants had stronger phenotypes than the *myb21-2* T-DNA insertion allele, which is in an intron and makes some full-length transcript (Table S1, Figure S4) [12]. Flowers of *myb24-2* and *myb24-5* single mutant plants appeared normal (Table S1, Figure 3E). Flowers of *myb21-5 myb24-5* double mutants grew similarly to *myb21-5* flowers up to stage 13 (Figure 3B, 3F, 3I; Table S1). However, whereas *myb21-5* flowers sometimes opened, *myb21-5 myb24-5* flower buds remained closed (Figure 3B, 3F). Moreover, *myb21-5 myb24-5* anthers failed to release pollen until after the flowers started to senesce, and treatment with exogenous MeJA failed to accelerate pollen release (Table S1).

### ARF6, ARF8, and MYB21 Promote Gynoecium Growth

As well as acting in petals and stamens, *MYB21* and *MYB24* are expressed in the gynoecium, suggesting that they may regulate aspects of gynoecium development or function. Gynoecia of wild-type, *arf6-2*, and *arf8-3* flowers grew to at least 4 mm long even if unpollinated (Figure S2A). Gynoecia of *arf6-2 arf8-3*, *myb21* and *myb21 myb24* flowers were shorter than wild-type gynoecia, and arrested at about 3 mm long (Table S1; Figure 3I, 3J; Figure S2A). This phenotype was largely attributable to decreased valve lengths in the mutants (Figure S6F). *myb21* mutations also decreased stigma lengths, although this effect was only statistically significant for both tested *myb21* alleles in *myb24-5* or *arf6-2/+ arf8-3* genetic backgrounds (Figure S6A–S6D, S6G). In the *arf6-2/+ arf8-3* genetic background, *myb21* mutations also decreased the proportion of ovules that were fertilized by wild-type pollen, from about 78% in *arf6-2*/*ARF6 arf8-3* ovules, to just 35–40% in *arf6-2*/*ARF6 arf8-3 myb21-4* or *arf6-2*/*ARF6 arf8-3 myb21-5* ovules. In many poorly fertilized gynoecia, pollen tubes only entered the apical part of the transmitting tract. These stigma and fertilization phenotypes were similar to, although less severe than, those observed for *arf6-2 arf8-3* plants (Figure S6E) [4].

### MYB21 Can Promote Petal Growth Independently of Jasmonate Response

Flowers of jasmonate-deficient (*aos-2*) or -insensitive (*coi1-1*) mutants had short stamens and indehiscent anthers similar to those of *myb21 myb24* mutants (Figure 3G, 3J; Figure S2C; Table S1). Similarly, at the time of wild-type flower opening (staged according to gynoecium length), *aos-2* and *coi1-1* flowers had delayed petal growth just as *myb21* and *myb21 myb24* flowers did, indicating that jasmonates promote petal growth at stage 12 (Figure 3G, 3J; Figure S2C). However, at stages 14–15 after pollination has normally occurred in wild-type flowers, petals of *aos-2* and *coi1-1* flowers continued to grow, so as to become larger than wild-type petals (Figure 3G, 3J; Figure S2C). Mutant flowers also senesced later than wild-type flowers, possibly accounting in part for the prolonged growth phase of these petals.

Gynoecia and valves of *aos-2* and *coi1-1* mutant flowers grew slightly less than those of unfertilized wild-type flowers, but more than those of *myb21* or *myb21 myb24* flowers (Figure 3J, Figure S2C, Figure S6F). Stigmas of *aos-2* and *coi1-1* flowers were as long as those of wild-type flowers, and *aos-2* and *coi1-1* gynoecia supported full fertilization after being pollinated manually (Figure S6G). Thus, *myb21* mutations had stronger effects on both petal and gynoecium growth than did *aos-2* or *coi1-1* mutations. The weaker phenotypes of *aos-2* and *coi1-1* than *myb21* and *myb21 myb24* mutants appears inconsistent with the hierarchical model in which jasmonates induce *MYB* genes which in turn cause petal expansion. These results might have arisen if the *aos-2* and *coi1-1* mutants each retain some jasmonate response. However, we detected no cis-JA in *aos-2* flowers (Figure 1F), and the *coi1-1* mutation is a null mutation in the only known JA-Ile receptor. Moreover, flowers of *aos-2 coi1-1* double mutant plants had enlarged petals and delayed senescence as did flowers of either single mutant (data not shown), suggesting that *aos-2* and *coi1-1* mutations each eliminated jasmonate response in flowers.

**Figure 4:**
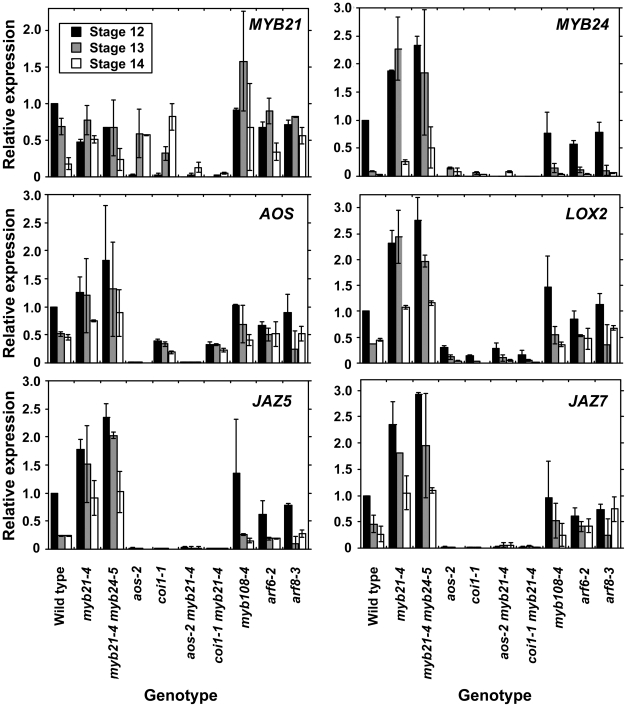
Expression of *MYB21*, *MYB24*, and jasmonate pathway genes in wild-type and mutant flowers at stages 12, 13, and 14. Gene expression was measured by quantitative RT-PCR. Shown are means of two biological replicates each having three technical replicates (± SD). Within each biological replicate, expression levels were normalized to expression in wild-type stage 12 flowers.

We therefore explored in more detail how the jasmonate pathway affects *MYB21* expression. In wild-type flowers, *MYB21* expression was high at stage 12, and then decreased at stages 13 and 14 (Figure 4, Table S3). Whereas at stage 12 *aos-2* and *coi1-1* flowers had lower expression of *MYB21* than did wild-type flowers, at stage 14 they had higher expression (Figure 4). Similarly, *aos-2 P_MYB21_:MYB21:GUS* plants had reduced X-Gluc staining at stage 13, but had X-Gluc staining in petals at stage 15 (Figure 2M, 2O). Thus, in both wild-type and jasmonate pathway mutant plants, petal growth correlated with *MYB21* expression. Moreover, petals of *myb21-4 aos-2*, *myb21-5 aos-2*, and *coi1-1 myb21-4* double mutant flowers failed to enlarge at late stages, and flower buds of these double mutants never opened (Table S1; Figure 3H, 3J; Figure S2C). Thus, MYB21 is active and promotes petal elongation in stage 14 *aos-2* and *coi1-1* flowers.

### Global Gene Expression Changes in Maturation-Deficient Flowers

**Figure 5:**
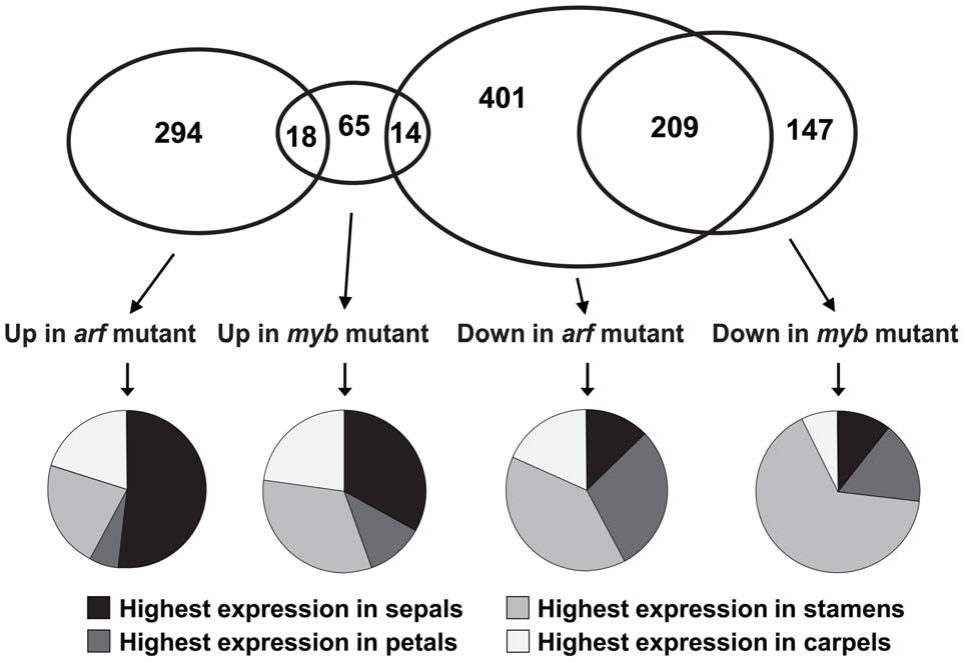
Global analyses of gene expression in *arf6-2 arf8-3* and *myb21-5 myb24-5* stage 12 flowers. Venn diagram indicates numbers of genes with higher or lower expression in mutant compared to wild-type flowers, based on a t-test (P<0.05) and a two-fold ratio of expression values. Pie charts indicate the proportion of genes in each expression class having highest expression in sepals, petals, stamens, or carpels of wild-type stage 12 flowers [21]. Table S3 lists these genes and provides details of their expression levels.

These analyses revealed that starting at flower stage 12, ARF6 and ARF8 promote *MYB21* and *MYB24* expression in multiple flower organs largely by increasing jasmonate levels. MYB21 and MYB24 in turn promote petal and stamen filament growth, anther dehiscence, and gynoecium growth and maturation, with MYB21 having a predominant role. To explore gene expression patterns underlying this regulatory hierarchy, we used Affymetrix ATH1 gene chip arrays to monitor global gene expression in wild-type, *arf6-2 arf8-3* and *myb21-5 myb24-5* closed buds (stage 12 flowers) and newly open flowers (stage 13). Expression data for each array probe set were compared statistically between genotypes, and in addition a two-fold expression ratio cutoff was applied to remove genes with statistically significant but small relative differences in expression level (Figure 5, Table S3). We focussed our analyses on gene expression changes at stage 12, when flowers of both double mutants have similar morphology to wild-type flowers. Stage 13 data are presented for reference (Table S3), but presumably include many indirect effects caused by developmental arrest of mutant flowers at stage 12. As most array probe sets correspond uniquely to a single gene, in the following analyses we refer to probe sets as “genes.”

At flower stage 12, 624 genes were expressed at a lower level in *arf6-2 arf8-3* flowers than in wild-type flowers, and 312 genes were expressed at a higher level (Figure 5). In *myb21-5 myb24-5* stage 12 flowers, 356 genes were underexpressed and 97 were overexpressed relative to wild-type flowers. Of the genes underexpressed in *arf6-2 arf8-3* flowers, 33% (209/624) were also underexpressed in *myb21-5 myb24-5* flowers, and 2% (14/624) were overexpressed. Of the genes overexpressed in *arf6-2 arf8-3* flowers, 6% (18/312) were also overexpressed in *myb21-5 myb24-5* flowers, and none was underexpressed. Thus, the *myb* mutations affected a greater proportion of genes that were underexpressed in *arf6-2 arf8-3* flowers than of genes that were overexpressed in *arf6-2 arf8-3* flowers. As MYB21 and MYB24 can activate genes [17], [18], the 209 genes underexpressed in both *myb21-5 myb24-5* and *arf6-2 arf8-3* flowers may include genes that the MYB proteins activate. Independent RNA blot hybridization and qRT-PCR experiments confirmed expression characteristics deduced from the array data for about 15 genes of interest (Figure 1, Figure 4, Figure S1C).

To discern patterns in the gene expression data, we compared our data to global gene expression datasets generated by other workers (Table S3). Gibberellins, acting in part through derepression of DELLA protein activity, also promote late stages of petal, stamen, and gynoecium development [14], [25]–[28]. We compared our gene expression results to a list of genes that were over- or under-expressed in *ga1-3* gibberellin-deficient mutant flowers [29]. 28% (172/624) of genes that were underexpressed in *arf6-2 arf8-3* flowers were also underexpressed in *ga1-3* flowers, and just 1.3% (8/624) were overexpressed in *ga1-3* flowers (Table S3). Similarly, 25% (77/312) of genes that were overexpressed in *arf6-2 arf8-3* flowers were also overexpressed in *ga1-3* flowers, and just 4.5% (14/312) were underexpressed in *ga1-3* flowers. Thus, ARF6 and ARF8 and gibberellin induce and repress an overlapping set of downstream responses in flowers, in most cases in the same direction.

We used data on gene expression in wild-type stage 12 sepals, petals, stamens and carpels [21] to determine in which organs each gene affected in *arf6-2 arf8-3* or *myb21-5 myb24-5* flowers was expressed (Figure 5, Table S3). Although most of the affected genes were expressed in multiple flower organs, to identify trends in the data it proved convenient to bin genes according to the organ in which they had highest expression in wild-type stage 12 flowers. Of the genes that were underexpressed in *arf6-2 arf8-3* flowers, substantial numbers were most highly expressed in sepals (79/624, 13%), petals (185/624, 30%), stamens (246/624, 39%) or carpels (114/624, 18%) of wild-type flowers. In contrast, of the genes that were overexpressed in *arf6-2 arf8-3* flowers, over half (161/312, 52%) were most highly expressed in sepals of wild-type flowers, whereas just 22% (69/312) were most highly expressed in wild-type stamens. In *myb21-5 myb24-5* flowers, 66% (234/356) of underexpressed genes had highest expression in stamens of wild-type flowers. Of the genes that were overexpressed in the *myb21-5 myb24-5* flowers, an equal number had highest expression in wild-type sepals as in wild-type stamens (32/97 in each case).

### Positive Feedbacks on Auxin Response and MYB Function

Among the genes with decreased expression in *arf6-2 arf8-3* flowers were several known auxin-inducible genes including *IAA1*, *SHY2/IAA3*, *IAA6*, *AXR2/IAA7*, *IAA17*, *IAA19*, *SAUR9* (*SMALL AUXIN UP RNA9*), *SAUR23*, *SAUR25*, *SAUR27*, *SAUR35*, *SAUR42*, *SAUR62-SAUR68*, and *SAUR70* (Table S3). Many of these have auxin response elements in their presumed promoters and are good candidates to be direct targets of ARF6 and ARF8 [30]. Although the hierarchical regulatory model does not predict that MYB21 or MYB24 should affect expression of direct ARF targets, several of these *IAA* and *SAUR* genes (*IAA6*, *IAA19*, *SAUR9*, *25*, *35*, *64*, *66*, *67*, and *68*) were also underexpressed in stage 12 *myb21-5 myb24-5* flowers (Table S3). RNA gel blot hybridization experiments confirmed that *IAA19* and *SAUR63* were underexpressed in *myb21-5 myb24-5* flowers, and that in addition *IAA2*, *SHY2/IAA3* and *AXR2/IAA7* were more modestly underexpressed in *myb21-5 myb24-5* flowers (Figure 1D). These results suggest that MYB21 and MYB24 participate in positive feedback loops that promote ARF activity.

Additional *ARF* and *MYB* genes were underexpressed in mutant flowers, and might also constitute positive feedbacks if they share targets with ARF6 and ARF8 or MYB21. The *ARF16* (*At4g30080*) gene encodes an Auxin Response Factor that is phylogenetically distant from ARF6 and ARF8, and regulates root cap differentiation together with its closest paralog *ARF10* [31], [32]. *ARF16* was underexpressed in *arf6-2 arf8-3* flowers but had normal expression level in *myb21-5 myb24-5* flowers. *arf10-3 arf16-2* flowers, as well as *P_35S_:MIR160c* flowers overexpressing a microRNA that targets *ARF10* and *ARF16* [31], had delayed stamen and petal growth, similarly to *arf6-2* or *arf8-3* single mutant flowers (Figure S2A, S2B). These results suggest that ARF10 and ARF16 act downstream of ARF6 and ARF8 to amplify stamen and petal growth at stage 12.

Analogously, *MYB57* and *MYB108*, closely related genes to *MYB21* and *MYB24*, were underexpressed in *arf6-2 arf8-3* and *myb21-5 myb24-5* flowers (Figure 1A, 1B; Table S3). A *P_MYB57_:MYB57:GUS* reporter was expressed in stamen filaments of opened wild-type flowers (Figure S1F). A *P_MYB108_:GUS* reporter was expressed in sepals and stamen filaments, particularly in the vasculature of these organs, and in the style (Figure S1G). The *myb57-1* mutant (Figure S3) had no obvious floral phenotypes (data not shown), although *myb57-1* can enhance a *myb21* mutation [14]. Flowers of *myb108* mutants (Figure S3) had normal organ lengths at stages 12 and 13, but had slightly delayed anther dehiscence (Table S1; Figure 3I; Figure S5E, S5F) [15]. In addition, *myb108-4* petals continued to grow after wild-type petals had stopped expanding, resulting in slightly longer petals at stage 14 (Figure 3I, Figure S5D–S5F). Similarly to the jasmonate pathway mutants, stage 14 *myb108-4* flowers had elevated *MYB21* expression, and *myb21-5 myb108-4* flowers had small petals (Figure 4, Table S1). Thus, increased *MYB21* expression may also cause persistent petal growth in *myb108* mutants.

### ARF6 and ARF8 Regulate Nectary Development

**Figure 6:**
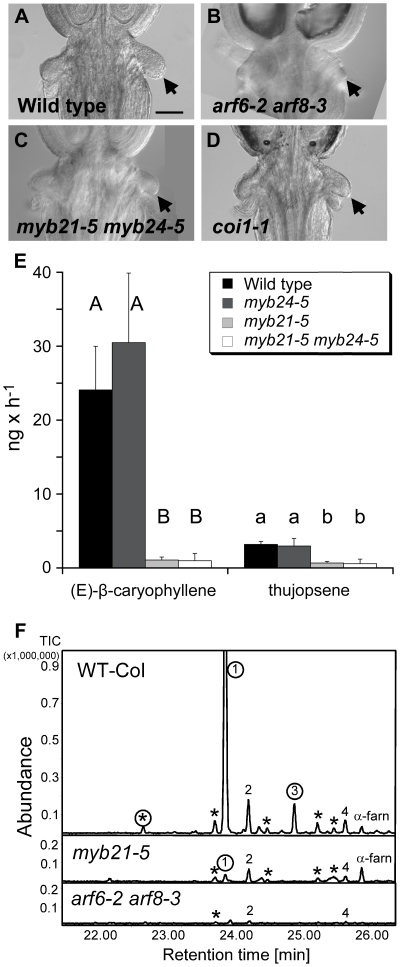
Phenotypes related to insect attraction. (A–D) Base of gynoecia of indicated genotypes. Arrows indicate nectaries. Scale bar, 0.1 mm. (E) Comparative quantitative analyses of floral volatile sesquiterpene emissions from wild-type, *myb21-5*, *myb24-5*, and *myb21-5 myb24-5* mutants. Emitted compounds were collected for 7 h from 40 detached inflorescences by a closed-loop stripping procedure. Emission was determined in ng h^−1^ per 40 inflorescences. Values are averages and standard deviations of three independent collections. Only emissions of (*E*)-β-caryophyllene, the product of TPS21, and thujopsene, the product of TPS11, are shown. Different letters indicate significant differences in emissions of each compound between genotypes ( p≤0.001). (F) GC-MS analyses of sesquiterpene hydrocarbons collected via SPME from 20 inflorescences of wild-type, *myb21-5* and *arf6-2 arf8-3* mutants. Peaks marked with circles represent sesquiterpenes produced by the terpene synthase TPS21. Compounds not labeled with circles are products of the terpene synthase TPS11, with the exception of α-farnesene (α-farn). 1, (*E*)-β-caryophyllene; 2, thujopsene; 3, α-humulene; 4, β-chamigrene. Peaks marked with asterisks are other sesquiterpene products of TPS11 or TPS21 [43].

*ARF6*, *ARF8*, *MYB21* and *MYB24* are each expressed in nectaries. A previous study identified 270 genes that were preferentially expressed in nectaries [33]. Of these, 18 were underexpressed in *arf6-2 arf8-3* only, 6 were underexpressed in *myb21-5 myb24-5* only, and 14 were underexpressed in both mutants (Table S3). In contrast, just 5 of the nectary-enriched genes were overexpressed in either mutant. Among the underexpressed genes were *CRABSCLAW* (*CRC/At1g69180*), which is required for nectary formation [34]; *YABBY5* (*At2g26580*) encoding a protein closely related to CRC; *CWIV4* (*At2g36190*) encoding a cell wall invertase required for nectary sink strength and nectar production [35]; *SWEET9* (*At2g39060*) encoding a nectary-specific glucose transporter [36]; and *JMT* (*At1g19640*) encoding S-adenosyl-L-methionine:jasmonic acid carboxyl methyltransferase, which makes the volatile compound methyl jasmonate [37]. Each of these genes was underexpressed in both *arf6-2 arf8-3* and *myb21-5 myb24-5* flowers, except for *CRC* which was underexpressed in *arf6-2 arf8-3* flowers only. Consistent with these gene expression changes, nectaries in *arf6-2 arf8-3* flowers were very small and only apparent by light microscopy in a fraction of flowers (Figure 6A, 6B). Nectaries in *coi1-1*, *arf6-2* and *arf8-3* single mutants and in *myb21-5 myb24-5* double mutant flowers appeared normal (Figure 6C, 6D; data not shown). These morphological and gene expression results indicate that ARF6 and ARF8 affect nectary growth and function, and that MYB21 and MYB24 affect nectary gene expression but not nectary formation.

### MYB21 Promotes Production of Volatile Sesquiterpenes

As flowers open, they emit volatile compounds, which may attract insect pollinators or predators, or may have a role in pathogen defense [38]–[41]. The Arabidopsis terpene synthase genes *TPS11* (*At5g44630*) and *TPS21* (*At5g23960*) synthesize a mixture of volatile sesquiterpenes emitted from flowers [42], [43]. Both genes were highly expressed in wild-type carpels, and *TPS11* was also expressed in nectaries [33], [43]. Both *TPS11* and *TPS21* were underexpressed in *arf6-2 arf8-3* and *myb21-5 myb24-5* flowers (Figure 1C, Table S3). Consistent with these patterns, *arf6-2 arf8-3* flowers emitted dramatically less sesquiterpenes produced by both TPS11 and TPS21 (Figure 6F). Similarly, *myb21-5* flowers had strongly reduced emission of sesquiterpenes produced by TPS21 (e.g. (*E*)-β-caryophyllene, α-humulene), and partially reduced levels of volatile sesquiterpenes produced by TPS11 (e.g. thujopsene, β-chamigrene) (Figure 6E, 6F). These effects are consistent with the gene expression patterns, as *TPS11* expression was reduced in *myb21-5 myb24-5* flowers by less than was *TPS21* expression (Table S3, Figure 1C). The *myb24-5* mutation did not affect emission of volatile sesquiterpenes, either by itself or in combination with *myb21-5* (Figure 6E). (*E*)-β-caryophyllene and thujopsene emissions were also reduced in flowers of the *opr3* jasmonate-deficient mutant (Figure S7).

### MYB21 and MYB24 Mediate Secondary Jasmonate Responses in Stamens

In a gene chip array dataset of gene expression in stamens of jasmonate-deficient *opr3* mutant stage 12 flowers treated with exogenous methyl jasmonate (MeJA), 31 genes were induced by at least 2-fold after 30 minutes of MeJA treatment, 179 additional genes were first induced after 2 hours, and 393 more genes were first induced after 8 hours [12]. *MYB21* and *MYB24* were themselves induced at the two hour time point in this dataset. Consistent with their reduced jasmonate production, *arf6-2 arf8-3* flowers underexpressed many of these MeJA-responsive genes, with the greatest proportional effect on the earliest MeJA-responsive genes. Thus, about 45% (14/31) of the genes induced by MeJA in stamens within 30 minutes were underexpressed in *arf6-2 arf8-3* flowers (Table S3).

In the *myb21-5 myb24-5* flowers, none of the early MeJA-inducible genes was underexpressed, and the proportion of MeJA-responsive genes affected was highest among those induced by MeJA at 8 hours. Of 86 late (8 hour) MeJA-inducible genes underexpressed in *arf6-2 arf8-3* flowers in our experiment, 50 (58%) were also underexpressed in *myb21-5 myb24-5* flowers, indicating that MYB21 and MYB24 mediate a large portion of late responses to jasmonate in flowers (Table S3).

### MYB21 Decreases Jasmonate Levels through a Negative Feedback Loop

Strikingly, 13 of the 14 genes that were underexpressed in *arf6-2 arf8-3* flowers but overexpressed in *myb21-5 myb24-5* flowers were MeJA-induced in stamens (Table S3). Using a less stringent 1.3-fold expression ratio cutoff, 71 genes were underexpressed in *arf6-2 arf8-3* flowers and overexpressed in *myb21-5 myb24-5* flowers, and 44 of these were MeJA-induced in stamens (13 of these at the earliest 0.5 h time point) (Table S4). Among these genes were *MYC2* (*At1g32640*), which binds to jasmonate-inducible promoters to mediate induction [44]; seven *JAZ* genes encoding negative regulators of jasmonate response [45]; and several genes encoding known or putative enzymes in the jasmonate biosynthesis pathway. These included *LOX2* (*At3g45140*) and *LOX4* (*At1g72520*) encoding lipoxygenases involved in generating the fatty acid precursor [46], [47]; *AOS* (*At5g42650*) encoding allene oxide synthase [48]; *OPR3* (*At2g06050*) encoding 12-oxophytodienoate reductase [8]; and *4CL11* (*At5g38120*) and *4CL9/OPCL1* (*At1g20510*), encoding 4-coumarate CoA ligases [49] (Table S4). RNA blot hybridization with polyA^+^ mRNA and qRT-PCR experiments confirmed increased expression of *LOX2* and *AOS* in *myb21-4 myb24-5* and *myb21-5 myb24-5* flowers (Figure 1E, Figure 4). The phospholipase *DAD1* (*At2g44810*) was expressed at a low level in all samples in the gene chip array experiment, but was also seen to be overexpressed in *myb21-5 myb24-5* flowers by RNA blot hybridization (Figure 1E). Consistent with their increased expression of jasmonate biosynthetic genes, stage 12–13 *myb21-5 myb24-5* flowers had about 12-fold higher level of cis-JA than did wild-type flowers (Figure 1F). The *AOS*, *LOX2*, *JAZ5*, and *JAZ7* genes were also overexpressed in *myb21-4* single mutant flowers, to the same degree as in *myb21-4 myb24-5* double mutant flowers (Figure 4). These data indicate that MYB21 acts within a negative feedback loop that regulates expression of multiple JA biosynthetic genes.

As mentioned above, the nectary-expressed *JMT* (*At1g19640*) gene whose product makes methyl jasmonate was underexpressed in both *arf6-2 arf8-3* and *myb21-5 myb24-5* flowers. However, the *At3g11480* gene encoding a JMT-related protein was overexpressed in *myb21-5 myb24-5* flowers, suggesting that *At3g11480* rather than *JMT/At1g19640* might produce MeJA as part of the MYB-regulated negative feedback loop. *JAR1* (*At2g46370*), encoding an enzyme that synthesizes the active JA-Ile, did not show statistically different expression between wild-type and mutant flowers. *jar1* plants are male-fertile, suggesting that another enzyme produces JA-Ile in flowers [50]. The most closely related Arabidopsis gene to *JAR1* is *GH3-10/DFL2* (*At4g03400*), which had normal expression in both mutants at stage 12, but was underexpressed in both mutants at stage 13 (data not shown).

In leaves, jasmonate induces genes encoding enzymes in the jasmonate biosynthesis pathway, indicating that a positive feedback loop amplifies jasmonate synthesis [7], [8], [47], [51]–[54]. In qRT-PCR assays, stage 12, 13, and 14 *aos-2* and *coi1-1* flowers had lower levels of *AOS* and *LOX2* than did wild-type flowers (Figure 4), confirming that such a positive feedback loop operates in flowers. To explore how the MYB21-mediated negative feedback and the COI1-mediated positive feedback interact, we assessed expression of these genes in *aos-2 myb21-4* and *coi1-1 myb21-4* double mutant flowers. In flowers of both double mutants, *AOS* and *LOX2* levels were as low as in *aos-2* or *coi1-1* mutant flowers. These results indicate that COI1 is required to activate jasmonate biosynthesis in *myb21-4* flowers, and suggest that MYB21 acts by inhibiting the COI1-mediated positive feedback loop in jasmonate biosynthesis. *AOS*, *LOX2*, *JAZ5*, and *JAZ7* were also underexpressed in *arf6-2 arf8-3 myb21-4* triple mutant flowers (Figure S1C), indicating that jasmonate overproduction in *myb21* mutant flowers also depends on ARF6 and ARF8.

## Discussion

### Functions of the Flower Maturation Regulatory Network

The phenotypic and gene expression analyses presented here show that, in addition to previously described petal, stamen, and gynoecium growth and maturation [2], [3], the ARF6 and ARF8 regulatory network promotes nectary development and floral scent production. This regulatory network should promote reproduction by both self-pollination and outcrossing. Thus, coordination of timing of stamen filament elongation, pollen release, stigma growth, and style and transmitting tract support of pollen tube growth ensures efficient self-fertilization; whereas coordination of petal growth, nectary development, and sesquiterpene production with stamen and gynoecium development would attract pollinators to flowers when they are reproductively competent. Although Arabidopsis self-pollinates efficiently, outcrossing by insect pollination has been observed in field populations [55], [56]. Terpene formation coordinated with gynoecium development also helps to protect reproductive organs against invasion by microbial pathogens (M. Huang, A. M. Sanchez-Moreiras, C. Abel, J. Gershenzon, and D. Tholl, unpublished results).

ARF6 and ARF8 activate jasmonate biosynthesis, which in turn activates *MYB21* and *MYB24*. Genes underexpressed in *arf6-2 arf8-3* and *myb21-5 myb24-5* flowers may promote aspects of flower maturation deficient in both mutants. Such genes include *MYB108*, which promotes anther dehiscence; several *SAUR* genes that promote organ elongation (K. Chae, C. G. Isaacs, P. H. Reeves, G. S. Maloney, G. K. Muday, and J. W. Reed, unpublished results); and the *TPS11* and *TPS21* genes required for sesquiterpene production. Genes affected in *arf6-2 arf8-3* but not *myb21-5 myb24-5* flowers must act upstream of MYB21 or mediate MYB21-independent functions. These include *ARF16*, which contributes to petal and stamen elongation; several genes involved in nectary formation or function; and three closely related bHLH transcription factors, *HALF-FILLED*(*HAF*)*/bHLH075*, *BRASSINOSTEROID ENHANCED EXPRESSION1* (*BEE1*)*/bHLH044* and *BEE3/bHLH050*, which act redundantly to promote transmitting tract differentiation (Table S3) [57], [58]. Other genes identified in this dataset may allow further dissection of general and organ-specific aspects of flower maturation, such as stylar factors that promote stigma growth non-cell-autonomously and/or potentiate pollen tube growth [22].

Sepal growth normally ceases at stage 12 when petal and stamen filament growth accelerates, and sepals of mutant flowers appeared outwardly normal. Nevertheless, 240 genes having preferential expression in wild-type sepals had altered expression in *arf6-2 arf8-3* flowers, and about two thirds (161/240) of these were overexpressed. In contrast, most affected genes with preferential expression in wild-type petals, stamens, or gynoecia were underexpressed in *arf6-2 arf8-3* flowers (91%, 78%, and 64%, respectively). Internal organs in the mutant flowers might have decreased sink strength, which might induce gene expression changes in sepals indirectly, or might cause internal organs to resemble sepals physiologically and express higher levels of “sepal” genes.

### Dynamic Interactions among Hormone Response Pathways during Flower Maturation

**Figure 7:**
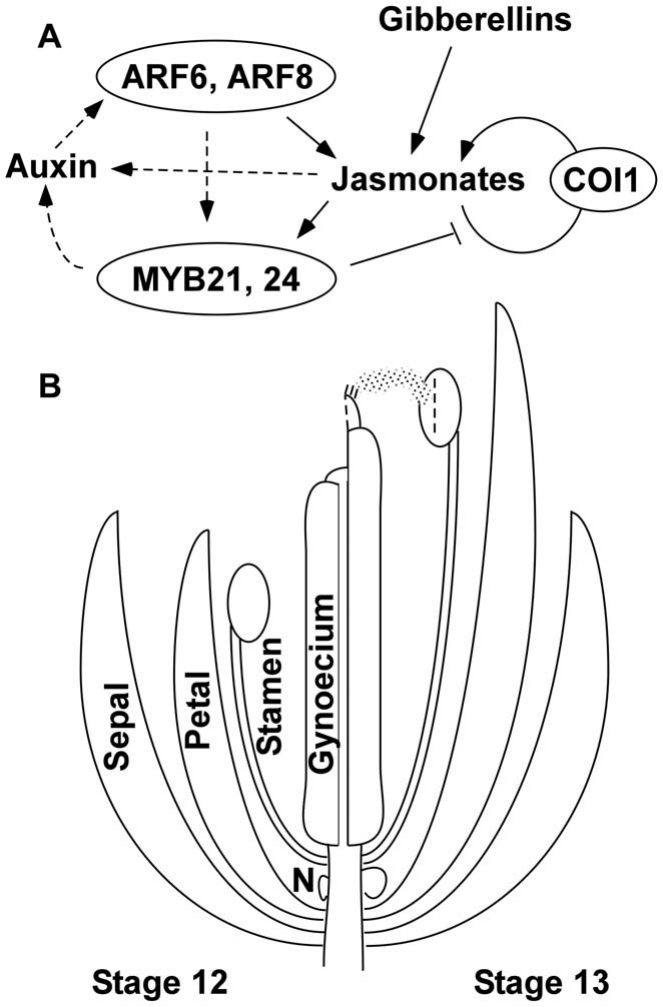
Genetic model of Arabidopsis flower maturation. (A) Diagram of principal regulatory pathways. Arrows indicate regulatory events established in this work or by previous studies. Both gibberellins and ARF6 and ARF8 auxin response factors promote jasmonate biosynthesis at flower stage 12. Auxin presumably enables ARF activity, and this may also be regulated by the circadian rhythm. Jasmonates in turn activate expression of genes for jasmonate biosynthesis, in a positive feedback loop requiring the JA-Ile receptor COI1. The underexpression of potential direct ARF6- and ARF8-targets in *myb21 myb24* flowers suggests that MYB21 and MYB24 may also participate in an additional positive feedback loop that promotes ARF6 and ARF8 activity, possibly through effects on auxin level (shown as dashed arrows). MYB21 represses jasmonate biosynthesis, and after the flower has opened (stage 13 and later), this negative feedback arrests flower maturation functions. In the absence of jasmonate signaling, ARF6 and ARF8 also contribute to *MYB21* expression in late-stage petals. (B) Illustration of flower developmental events regulated by the network between flower stage 12 (left) and stage 13 (right). The network induces downstream effectors that promote multiple events including petal and stamen filament elongation (regulated by ARF16 and by SAUR proteins), anther dehiscence (regulated by MYB108), volatile compound production (by TPS11 and TPS21 terpene synthases), nectary growth and development (regulated by CRC), and gynoecium growth and maturation. These and other effector genes may be activated directly or indirectly by MYB21 and MYB24, or by ARF6 and ARF8 independently of the MYB proteins. N, nectary.

Three mobile hormone signals - auxin, gibberellin, and jasmonate - regulate flower maturation, and this network incorporates both signal amplification and feedback mechanisms (Figure 7). Auxin can activate ARF6 and ARF8 activity by destabilizing Aux/IAA transcriptional repressor proteins, and both *msg2*/*iaa19* gain-of-function mutants and *yucca2 yucca6* mutants deficient in auxin biosynthesis have delayed stamen development [23], [59]–[64]. These results indicate that auxin indeed promotes wild-type flower maturation. Temperature stress, shade light and the circadian rhythm can each regulate auxin levels and/or response [65]–[71], and these environmental factors might thereby regulate flower growth according to light or temperature conditions, or ensure appropriate diurnal timing of flower opening and pollination.

Similarly to *arf6 arf8* mutants, gibberellin-deficient mutants have arrested petal, stamen, and gynoecium development, are deficient in jasmonate production, and are both male- and female-sterile [14], [26], [28]. Although the two pathways had overlapping effects on gene expression, based on our gene chip expression data, *arf6-2 arf8-3* flowers had normal gibberellin biosynthetic gene expression levels, and known auxin biosynthetic genes did not appear in the gibberellin-responsive gene lists. Thus, the two pathways may be integrated through shared downstream targets rather than acting hierarchically. Auxins and gibberellins also each regulate hypocotyl elongation and fruit growth, by both hierarchical and parallel mechanisms [72]–[75].

ARF6 and ARF8 and gibberellins each activate jasmonate biosynthesis. ARF6 and ARF8 may do this in part through *TCP4* (*At3g15030*), which was underexpressed in *arf6-2 arf8-3* flowers and activates developmental expression of *LOX2* [76]. JA-Ile in turn activates a positive feedback loop of jasmonate synthesis by causing COI1-dependent turnover of JAZ transcriptional repressor proteins, which then (at least in leaves) releases the bHLH proteins MYC2, MYC3, and MYC4 to activate transcription of jasmonate biosynthesis genes as well as *MYC2* itself [77]–[79]. Jasmonate synthesis has been postulated to occur in stamen filaments, based on the expression pattern of *DAD1* [6], [80]. However, other genes can act redundantly with DAD1 during wound-induced jasmonate production [81], and other jasmonate biosynthetic genes were expressed in multiple flower organs (Table S3) [21], [47], suggesting that jasmonates are synthesized broadly throughout the flower. If synthesis were first triggered in stamen filaments, the positive feedback of jasmonate synthesis and movement of MeJA or another jasmonate pathway compound might amplify jasmonate production throughout the flower, thereby causing a coordinated burst of stamen and petal growth and emission of floral scents.

Jasmonates induce *MYB21* and *MYB24*, and MYB21 and MYB24 then activate secondary gene expression responses to jasmonate leading to petal and stamen filament elongation and anther dehiscence. MYB21 and MYB24 are also required for expression of several known primary auxin-responsive genes. This finding suggests that MYB21 and MYB24 also affect ARF6 and ARF8 activity, and that a portion of the *myb21 myb24* flower phenotypes may be caused by decreased ARF activity.

MYB21 also induces a negative feedback on jasmonate biosynthesis. Jasmonate overproduction in *myb21* flowers requires the COI1-dependent positive feedback pathway that activates jasmonate biosynthesis genes, suggesting that MYB21 acts on a component of this pathway. *JAZ* genes encoding repressors of jasmonate response are themselves jasmonate-inducible, and the MYB proteins might amplify this negative feedback loop if they activate *JAZ* gene expression. However, the increased rather than decreased expression of *JAZ* and other primary jasmonate responsive genes in *myb21-5 myb24-5* flowers suggests that other proteins such as MYC2 are sufficient to activate primary jasmonate response. Alternatively, as suggested by the recent discovery that MYB21 and MYB24 proteins can interact with JAZ1, JAZ8, JAZ10, and JAZ11 proteins [20], MYB21 might stabilize JAZ proteins by interfering with their COI1-mediated turnover. This negative feedback pathway may also act in flowers of the *jar1-1* mutant deficient in the enzyme that makes active JA-Ile, which similarly had elevated jasmonic acid levels [82].

In wild-type flowers, jasmonic acid levels increase at stages 11–12 just before flowers open, and then decrease at stages 13–14, when flower organs stop growing [2]. Mathematical modeling suggests that after wounding of leaves, positive feedback increases the amplitude of jasmonate synthesis, whereas negative feedback mediated by the JAZ proteins determines the duration of the jasmonate pulse [83]. In flowers, the linked positive and negative feedback loops regulating jasmonate production and auxin response provide a plausible mechanism for inducing coordinated rapid increase in petal and stamen growth at stage 12, followed by a quick cessation of growth after stage 13 once the flower has opened and pollen has been released. MYB21 and MYB24 are not expressed in wounded leaves, and recruitment of the MYB factors into the feedback mechanisms may be an evolutionary innovation that has contributed to the adaptation of this network to regulate flower opening.

The prolonged growth seen in petals of stage 13–14 jasmonate pathway mutant flowers arises from jasmonate-independent *MYB21* expression. As *arf6 arf8* flowers do not express *MYB21*, an ARF-dependent but jasmonate-independent mechanism can apparently activate *MYB21*. This or a similar pathway apparently also acts in stage 14 *arf8* and *myb108* mutant flowers [11]. *BIGPETAL* (*BPE*)/*bHLH31* (*At1g59640*) is activated in petals by jasmonate-induced alternative splicing and represses petal growth [9], [10], [12], [58], and it will be interesting to test whether it represses *MYB21*.

### Action of this Network in Other Angiosperms

In tobacco and petunia, putative orthologs of *MYB21* and *MYB24* regulate both floral scent production and flower opening [84]–[86]. The network described here may thus provide a useful context to understand flower maturation in other angiosperms, and the roles of genes responsible for natural variation in flower morphology [87]. For example, variation in the expression level of the tomato *Style2.1* gene determines the extent of style growth, which in turn affects whether the plant self-pollinates or outcrosses [88]. An Arabidopsis homolog of *Style2.1*, *PRE1/bHLH136/BNQ1* (*At5g39860*), is underexpressed in *arf6-2 arf8-3* flowers and may contribute to Arabidopsis flower organ elongation [89], [90].

## Materials and Methods

### Plant Material and Isolation of *arf6-2* Enhancer Mutants

All genotypes were in the Columbia ecotype of *Arabidopsis thaliana*. *arf6-2* and *arf8-3* mutants were previously described [2]. The *myb21-4* and *myb21-5* mutants were isolated from an EMS mutagenesis screen for enhancers of the *arf6-2* mutant. *arf6-2* seeds were treated with 0.2% EMS for 16 hours, and 10,000 M2 plants derived from approximately 5000 M1 parents were screened for reduced fecundity. In addition to the *myb21* mutations described here, we isolated three new *arf8* alleles in this screen (Table S2). *arf6-2 myb21* plants from the screen were back-crossed once to *arf6-2*, and then crossed twice to wild type prior to further analysis. Backcrosses indicated that the *myb21* phenotype was caused by a recessive mutation at a single genetic locus. To map the mutations, *arf6-2 enhancer* mutants were crossed to an *arf6-2* line that had been introgressed into the Landsberg *erecta* ecotype. A bulked-segregant analysis approach using 29 markers evenly distributed over the genome was used to establish a preliminary map position [91], and the map position was then refined using closely linked SSLP, CAPS and dCAPS markers (Figure S3).

T-DNA insertion mutations in *MYB21*, *MYB24*, *MYB57*, *MYB108*, and *AOS* from the SALK Genomic Analysis Laboratory were provided by the Arabidopsis Biological Resource Centre [92]. Homozygous mutants were identified within segregating T3 and T4 populations. Details on these mutants, and PCR primers used to identify mutant alleles, are provided in Figure S4, Table S2, and Table S5. Double and triple mutants were identified in the F2 progeny of crosses from the respective single or double mutant parents. Most genotypes were fertile when manually self-pollinated. However, *myb21 myb24*, *myb21 aos-2* and *myb21-5 arf6-2 arf8-3* plants were maintained as *myb21/+ myb24*, *myb21 aos-2/+* and *myb21 arf8-3 arf6-2/+* stocks. *coi1-1* seeds were provided by John Turner (University of East Anglia, Norwich, UK), and *P_LAT52_:GUS* seed were provided by Mark Johnson (Brown University, Providence, RI). *ams* seeds (SALK_152147) [93] were provided by Hong Ma (Pennsylvania State University, College Station, PA).

### Phenotypic Analyses

To measure flower organs across a developmental series, flower buds were dissected, and flower organs were placed on an agar plate and measured using a camera lucida attachment on a dissecting microscope. For measurements of floral organ lengths and timing of anther dehiscence in Table S1, the first open flower of wild-type plants was designated as flower 1 (stage 13) [1]. For genotypes in which flower opening was impaired, equivalent stage flowers were identified based upon bud size and position on the inflorescence stem. Scanning electron microscopy was carried out as previously described [2]. Fertilization frequencies were assessed by X-Gluc staining 24 hours after pollination with the pollen-specific reporter line *P_LAT52_:GUS* [94]. In these assays, 87% or more of wild-type, *ams* male-sterile, *myb21-5*, *myb21 myb24*, and *myb21 myb24 myb108* ovules were fertilized, as judged by strong X-Gluc staining in ovules in which a pollen tube had ruptured.

### Transgenic Plants

To make GUS reporter constructs, promoter and genomic sequences lacking the endogenous stop codon were cloned into the Gateway pENTR/D-TOPO vector (Invitrogen Life Technologies, Carlsbad, CA). The upstream region (2266 bp) and first exon of *MYB21* were amplified by PCR using the primers MYB21 PF and MYB21 R4 (Table S5). The introns and second and third exons were amplified using the primers MYB21 F4 and MYB21 R7. These two PCR products were cloned separately into pENTR/D-TOPO, and then the promoter and first exon of *MYB21* were excised and ligated into the construct containing the 3′end of the *MYB21* gene using *Not*I and *Pst*I. The upstream region (2207 bp) and first exon of *MYB24* was amplified by PCR using the primers MYB24 PF and MYB24 R3. The entire predicted coding region of *MYB24* was amplified using the primers MYB24 F2 and MYB24 R2. These two PCR products were cloned separately into pENTR/D-TOPO, and then the promoter and first exon were excised and ligated into the construct containing the 3′end of *MYB24* gene using *Not*I and *Pst*I. The *MYB57* upstream region (2414 bp) and predicted coding region were amplified using the primers MYB57 PF and MYB57 R2 and cloned into pENTR/D-TOPO. For *MYB108*, only the promoter was used in the GUS reporter construct. The upstream region of *MYB108* (2090 bp) was amplified using the primers MYB108 PF and MYB108 R2 and cloned into pENTR/D-TOPO. The promoter and genomic sequences were fused to the GUS reporter by recombining entry clones into the destination vector pGWB3 [95] using LR clonase (Invitrogen). Transformation of Arabidopsis plants and histochemical staining were performed as described previously [96], [97]. The *P_MYB21_:MYB21:GUS* and *P_MYB24_:MYB24:GUS* constructs partially rescued the phenotype of *myb21-2 myb24-2* flowers, indicating that they retained some MYB21 and MYB24 activity.

For *P_35S_:MYB21* and *P_35S_:Green fluorescent protein(GFP):MYB21* constructs, a genomic *MYB21* fragment was amplified using the primers MYB21 GWF and MYB21 GWR* (Table S5), and cloned into the Gateway pENTR/D-TOPO vector. For *P_35S_:MYB21*, the entry clone was recombined into pB2GW7 [98]. For *P_35S_:GFP:MYB21* the entry clone was recombined into pGWB6 [95]. Transgenic T1 *P_35S_:MYB21* and *P_35S_:GFP:MYB21* plants showed a range of phenotypes, including narrow leaves, dwarfism, and floral defects, similar to those previously described [19], [20]. For our analyses, we used weaker lines that had less severe phenotypes.

### Hormone Treatments

For gene expression analyses, plants were sprayed with 1 mM MeJA (Bedoukian Research, Inc, Danbury, CT) or 10 µM IAA (Sigma) in 1% methanol 0.05% Tween-20, or with solvent alone, and were harvested after two hours (MeJA) or the specified time periods (IAA). To restore fertility to JA-deficient plants and to assess the effect of jasmonate on *aos-2 P_MYB21_:MYB21:GUS* plants, flowers were sprayed with 1 mM MeJA daily for 4 days.

### Gene Expression Analyses

Flowers or whole inflorescences were frozen in liquid N_2_ and total RNA was isolated using Trizol reagent (Invitrogen Life Technologies, Carlsbad, CA) or with RNeasy plant mini kits (Qiagen). Poly (A^+^) RNA was extracted from 50 µg of total RNA using oligo(dT)_25_ Dynabeads according to manufacturers' instructions (Dynal A.S., Oslo, Norway). RNA gel blot hybridizations were performed as described [99]. Probes were created by PCR using genomic DNA or Peking-Yale cDNA clones (Arabidopsis Biological Resource Center) as template using primer pairs listed in Table S5.

For real-time quantitative RT-PCR analyses, total RNA was extracted from stage 12, 13 and 14 flowers in the morning between 2 and 4 hours after subjective dawn. cDNA was synthesized using the Reverse Transcription System (Promega A3500) with random primers according to the manufacturer's instructions. 0.1 µg of total RNA was used for the 20 µl volume reaction and incubated for 1 hr at 42°C. The RT reaction mixture was diluted 10-fold and 1 µl was used as a template in 10 µl PCR reactions using the Applied Biosystems real-time PCR systems in standard mode with SYBR Green Master Mix (Applied Biosystems) following the manufacturer's protocol. The primers used for qRT-PCR analysis are listed in Table S5. Reactions were performed in triplicate and the products were checked by melting curve analysis. Transcript levels were normalized to the level of reference transcript *UBQ10*.

For Affymetrix gene chip gene expression analyses, RNA was isolated from stage 12 (largest closed buds) and stage 13 (first open flowers) harvested in the morning between 2 and 4 hours after subjective dawn. Three biological replicates were performed. Probe synthesis and gene chip hybridizations were performed by the UNC-CH Functional Genomics Core Facility. Total RNA (1000 ng) was used to synthesize cDNA followed by aRNA. The MessageAmp II-Biotin Enhanced Kit (Ambion) was used to generate biotinylated aRNA from the cDNA reaction. The aRNA was then fragmented in fragmentation buffer from the Ambion kit at 94°C for 35 minutes before the chip hybridization. Fragmented aRNA (15 µg) was then added to a hybridization cocktail (0.05 µg/µl fragmented aRNA, 50 pM control oligonucleotide B2, *BioB*, *BioC*, *BioD* and *cre* hybridization controls, 0.1 mg/ml herring sperm DNA, 0.5 mg/ml acetylated BSA, 100 mM MES, 1 M [Na+], 20 mM EDTA, 0.01% Tween 20). aRNA (10 µg) was used for hybridization in a volume of 200 µl per slide. ATH1 arrays [100] (Affymetrix, Santa Clara, CA) were hybridized for 16 hours at 45°C in the GeneChip Hybridization Oven 640 (Affymetrix). The arrays were washed and stained with R-phycoerythrin streptavidin in the GeneChip Fluidics Station 450 (Affymetrix) using wash protocol EukGE-WS2v4, and arrays were scanned with the GeneChip Scanner 3000 7G Plus with autoloader. Affymetrix MAS 5.0 GeneChip Operating Software was used for washing, scanning and basic analysis. Sample quality was assessed by examination of 3′ to 5′ intensity ratios of selected genes. Data were analyzed using Genespring GX 10.0.1 software. Raw data were background corrected and normalized using the RMA algorithm with no baseline correction. Means for each gene over the three biological replicates were calculated, and statistical differences between wild-type and mutant expression levels assessed by t-test without multiple testing correction. Genes reported in Table S3 are those with P<0.05 and having 2-fold or greater expression level difference from the corresponding wild-type sample. Gene chip hybridization data have been deposited in the NCBI GEO database (http://www.ncbi.nlm.nih.gov/geo/) with accession number GSE32193.

### *In Situ* Hybridization

*In situ* hybridizations were carried out as previously described [4]. *MYB21* and *MYB24* probes were PCR amplified from genomic DNA using primers that spanned the last exon (MYB21 ins-HindIII F, MYB21 ins-BamHI R; MYB24 ins-HindIII F, MYB24 ins-BamHI R) (Table S5). PCR products were then cloned into the pGEM-T vector (Promega). *MYB21* and *MYB24* sense probes produced no signal in wild-type flowers.

### Jasmonic Acid and Volatile Sesquiterpene Collection and Analysis from Flowers

Jasmonic acid was measured as described [101] from stage 12–13 flowers collected in the morning and frozen in liquid nitrogen. To measure sesquiterpenes, volatile compounds were collected in 1 L bell jars with 40 detached inflorescences placed in a small glass beaker filled with tap water, under controlled temperature and light conditions (22°C, 150 µmol m^−2^ s^−1^ PAR). Emitted volatile compounds were collected for 7 h on 5 mg Charcoal filter traps (Gränicher and Quartero, Daumazan, France) in a closed-loop stripping procedure [102] and then eluted from the traps with 40 µl CH_2_Cl_2_ containing 1-bromodecane (20 ng/µl) as a standard. Sample analysis and quantification of terpenes was performed by gas chromatography–mass spectrometry (GC-MS) on a Shimadzu QP 2010s GC-MS instrument as described previously [103]. Separation was performed on a (5%-phenyl)-methylpolysiloxane (DB5) column (Restek, 30 m×0.25 mm i.d.×0.25 m –thickness). Helium was the carrier gas (flow rate 1.4 ml min^−1^), a splitless injection (injection volume 1 µl) was used, and a temperature gradient of 5°C/min from 40°C (2 min hold) to 220°C was applied. Compounds were identified by comparison of retention times and mass spectra with those of authentic standards. Trapping and GC-MS analysis of volatiles from flowers of *opr3* and Wassilewskija wild type were performed as described in [43]. Statistical significance of differences in volatile emission was determined with SAS9.1 (SAS Institute Inc., Cary, NC, USA) using student's t-test or ANOVA with Tukey post-hoc test.

For an alternative fast sampling and analysis of volatile compounds, 20 inflorescences were placed in a 20 ml screw cap glass vial containing 4 ml of water. Inflorescences were incubated in the sealed vial for 2 h under the conditions described above. Volatile compounds were then trapped by solid phase microextraction (SPME) for 30 min at 40°C and injected into the GC by thermal desorption using an automated SPME sampling device (Combi-PAL, CTC Analytics, Zwingen, Switzerland).

### Gene Accession Numbers

Arabidopsis Genome Initiative locus identifiers for the genes studied in this article are as follows: *AOS* (At5g42650); *ARF6* (At1g30330); *ARF8* (At5g37020); *COI1* (At2g39440); *IAA2* (At 3g23030); *IAA3* (At1g04240); *IAA4* (At5g43700); *IAA7* (At3g23050); *IAA13* (At2g33310); *IAA16* (At3g04730); *IAA19* (At3g15540); *MYB21* (At3g27810); *MYB24* (At5g40350); *MYB57* (At3g01530); *MYB108* (At3g06490); *OPR3* (At2g06050); *SAUR63* (At1g29440); *AtTPS11* (At5g44630); *AtTPS21* (At5g23960).

## Supporting Information

Figure S1 Gene expression in mutant and transgenic flowers, and expression of *MYB* reporter lines. (A) RNA gel blot hybridization with *MYB21*, *MYB24* and *ARF6* probes. RNA from wild-type, *arf6-2 arf8-3* and two independent *P_35S_:ARF6* lines [4]. A *β-tubulin* probe was used as a loading control. The *ARF6* transcript is smaller in *P_35S_:ARF6* lines than in wild type, because the transgene lacks the endogenous 5′ and 3′UTR sequences. (B) *arf6-2 arf8-3 P_MYB21_:MYB21:GUS* stage 13 flower stained with X-Gluc. (C) Quantitative RT-PCR assays of expression of indicated genes in pooled stage 12–13 flowers of indicated genotypes. Shown are means of two biological replicates each having three technical replicates (± SD). Within each biological replicate, expression levels were normalized to expression in wild-type flowers. (D–F) X-Gluc-stained stage 14 flowers of *P_MYB21_:MYB21:GUS* (D), *P_MYB24_:MYB24:GUS* (E), *P_MYB57_:MYB57:GUS* (F), and *P_MYB108_:GUS* (G). Inset in (E) shows expression in nectaries.
(TIF)

Figure S2 Petal and stamen lengths relative to gynoecium length for individual flowers. (A) Wild-type, *arf6-2*, *arf8-3*, and *arf6-2 arf8-3* flowers. (B) Wild-type, *arf10-3 arf16-2*, and *P_35S_:MIR160c* flowers. The microRNA *miR160* targets both *ARF10* and *ARF16*. (C) Wild-type, *coi1-1*, *myb21-4*, and *coi1-1 myb21-4* flowers.
(TIF)

Figure S3 Map-based cloning of *MYB21*. (A) Map-based cloning of *MYB21*. Bulked segregant analysis was used to establish linkage of the *arf6* enhancer (*myb21-5*) to marker *ciw11a* on chromosome 3. 355 *arf6-2 myb21-*like F2 plants from an *arf6-2 myb21-5*×*arf6-2* (La-*er*) cross were then screened with PCR-based markers closely linked to *ciw11a*. One crossover event was detected between *myb21-5* and each of the markers MGF10-40054 and K16N12-45751, indicating that the *myb21-5* mutation was located within a 91 kb interval between these two markers. Sequencing of the *MYB21* gene in this interval identified premature stop codons in both *myb21-4* and *myb21-5* alleles. (B) Alignment of predicted MYB21 and MYB24 amino acid sequences. Identical residues are shaded black, similar residues are shaded grey. Asterisks (*) indicate positions of the *myb21-4* and *myb21-5* point mutations. (C) Photographs of stage 13 flowers of wild type, *myb21-5* and three independent *myb21-5 P_35S_:GFP:MYB21* lines. The *P_35S_:GFP:MYB21* transgene can restore petal and stamen elongation to the *myb21-5* plant, and rescue the anther dehiscence defect. The flowers shown are the first open flower on wild-type inflorescence (stage 13, flower position 1), or its equivalent based upon bud size and position on the inflorescence stem.
(TIF)

Figure S4 Mutations in *MYB21*, *MYB24*, *MYB108*, *MYB57* and *AOS*. (A) Positions of mutations in *MYB21*, *MYB24*, *MYB108*, *MYB57* and *AOS* genes. Exons are shown as blue boxes, T-DNA insertions are shown as triangles. The positions of T-DNA insertions are based upon sequencing provided by SIGNAL database [92]. (B) RNA gel blot hybridizations using *MYB21*, *MYB24*, *MYB108*, *MYB57* and *AOS* probes. RNA was isolated from flowers of wild-type and homozygous T-DNA insertion mutant plants. A *β-tubulin* probe was used as a loading control. The transcript in *myb21-2* flowers was confirmed to be *MYB21* by sequencing RT-PCR products from the mutant. In some blots, the *myb24-5* mutant had a transcript of a larger size, possibly arising from fusion to T-DNA sequences, that is not shown in the figure.
(TIF)

Figure S5 *myb21-5* and *myb108-4* flower phenotypes. (A–C) Scanning electron micrographs of stage 12 and 13 wild-type flowers (A,C) and stage 13 *myb21-5* flower (B). For each picture, the left panel shows a flower with intact gynoecium and stamens (and perianth organs removed), and the right panel shows a closeup of the stamen filament. Scale bars: 800 µm (left panels); 100 µM (right panels). (D) Photographs of *myb108-4* inflorescence and stage 13 flower. Asterisk in left panel indicates first open flower. (E,F) Closeup photographs of stage 14 wild-type and *myb108-4* flowers, showing failure of anther dehiscence in the mutant.
(TIF)

Figure S6 Gynoecium phenotypes of *myb21* and *arf6 arf8* mutants. (A–E) Scanning electron micrographs of stigmas from (A) wild-type (stage 13), (B) *myb21-5* (stage 13), (C) *arf6-2/ARF6 arf8-3* (stage 14), (D) *arf6-2/ARF6 arf8-3 myb21-5* (stage 14), and (E) *arf6-2 arf8-3* (stage 14) flowers. Scale bar = 200 µm. (F) Lengths of valves of stage 13–14 flowers of indicated genotypes. (G) Lengths of styles and stigmas of stage 13–14 flowers of indicated genotypes. Values in F and G are means ± SD for between 8 and 50 measurements. * indicates difference from corresponding wild-type measurement by *t*-test with P<0.001; † indicates significant difference from value for corresponding *MYB21^+^* genotype by *t*-test with P<0.001. In A, F and G, wild-type flowers were emasculated 2-3 days before measurements, to prevent pollination and subsequent stigma collapse.
(TIF)

Figure S7 Volatile sesquiterpene emissions from wild-type and *opr3* inflorescences. Emissions of (*E*)-β-caryophyllene and thujopsene are shown. Volatile compounds were collected from 70 inflorescences for 9 h by a closed-loop stripping procedure. The wild-type and *opr3* plants were in the Wassilewskija (Ws) ecotype. Student's t-test,***, p<0.001; **, p<0.01.
(TIF)

Table S1 Floral organ lengths in single and higher-order mutants, and timing of pollen release. The first open flower on wild-type inflorescence was designated flower 1 (stage 13), and older flowers numbered sequentially. For genotypes with delayed flower opening, the equivalent flower was chosen based upon bud size and position on the inflorescence stem. For MeJA treatments inflorescences were sprayed once a day with 1 mM MeJA for four days before taking measurements. Data from three experiments are shown. n = 10, data is shown as mean ± standard deviation. *Pollen release in these genotypes occurred as the floral organs senesced.
(XLS)

Table S2 Genotyping details for mutant alleles used in this study. Mutations for which genotyping information has been reported previously are not included here. The positions of T-DNA insertions are based on end-sequencing provided by the SIGNAL database [92]. Primer sequences are provided in Table S5.
(XLS)

Table S3 Genes with increased or decreased expression in *arf6-2 arf8-3* or *myb21-5 myb24-5* stage 12 flowers, by at least two-fold relative to wild-type flowers. Listed are genes whose expression at stage 12 was statistically different from wild-type expression and that differed by at least 2-fold from wild type. Columns from left to right: (A,I) Affymetrix ATH1 gene id; (B–G) expression in wild-type stage 12 flowers and flower organs [21]; (H) genes with enriched expression in nectaries [33]; (J–O) Gene expression levels in wild-type, *arf6-2 arf8-3*, and *myb21-5 myb24-5* stage 12 and stage 13 flowers, averaged from three replicates (this work); (P) Genes with altered expression in *ga1-3* flowers [29]; (Q) genes induced in stamens by MeJA [12]; (R–T) gene annotation and AGI number. To aid in reading the table, cells in some columns are color-coded to indicate genes with particular expression characteristics, according to the keys listed below the corresponding columns. The data may be sorted in Excel to group genes with common expression characteristics.
(XLS)

Table S4 Genes underexpressed in *arf6-2 arf8-3* and overexpressed in *myb21-5 myb24-5* stage 12 flowers, by at least 1.3-fold relative to wild-type flowers. Columns: (A,H) Affymetrix ATH1 gene id; (B–G) expression in wild-type stage 12 flowers and flower organs [21]; (I–N) Gene expression levels in wild-type, *arf6-2 arf8-3*, and *myb21-5 myb24-5* stage 12 and stage 13 flowers, averaged from three replicates (this work); (O) genes induced in stamens by MeJA [12]; (P–R) gene annotation and AGI number. To aid in reading the table, cells in some columns are color-coded to indicate genes with particular expression characteristics, according to the keys listed below the corresponding columns.
(XLS)

Table S5 Oligonucleotide primers used in this study. For some primers, a CACC sequence was incorporated at the 5′ end to enable directional cloning into the pENTR/D-TOPO vector.
(XLS)
