# Supplementary figures and images for: A Regulatory Network for Coordinated Flower Maturation
Source: PLoS Genet. 2012 Feb 9;8(2):e1002506. doi: 10.1371/journal.pgen.1002506 (PMC3276552; doi:10.1371/journal.pgen.1002506)

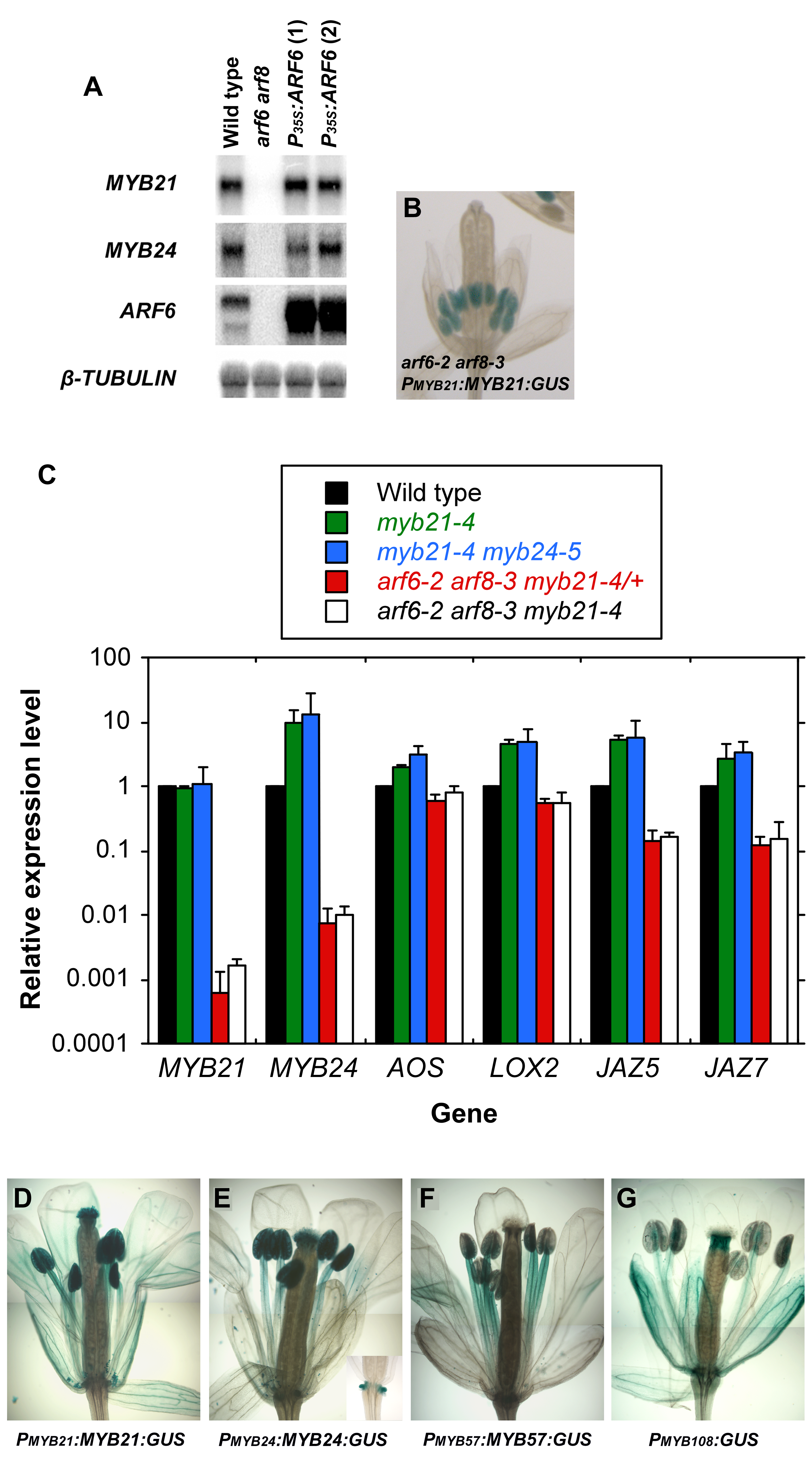

Supplement: Figure S1 — Gene expression in mutant and transgenic flowers, and expression of MYB reporter lines. (A) RNA gel blot hybridization with MYB21, MYB24 and ARF6 probes. RNA from wild-type, arf6-2 arf8-3 and two independent P35S:ARF6 lines [4]. A β-tubulin probe was used as a loading control. The ARF6 transcript is smaller in P35S:ARF6 lines than in wild type, because the transgene lacks the endogenous 5′ and 3′UTR sequences. (B) arf6-2 arf8-3 PMYB21:MYB21:GUS stage 13 flower stained with X-Gluc. (C) Quantitative RT-PCR assays of expression of indicated genes in pooled stage 12–13 flowers of indicated genotypes. Shown are means of two biological replicates each having three technical replicates (± SD). Within each biological replicate, expression levels were normalized to expression in wild-type flowers. (D–F) X-Gluc-stained stage 14 flowers of PMYB21:MYB21:GUS (D), PMYB24:MYB24:GUS (E), PMYB57:MYB57:GUS (F), and PMYB108:GUS (G). Inset in (E) shows expression in nectaries. (TIF) [file pgen.1002506.s001.tif]

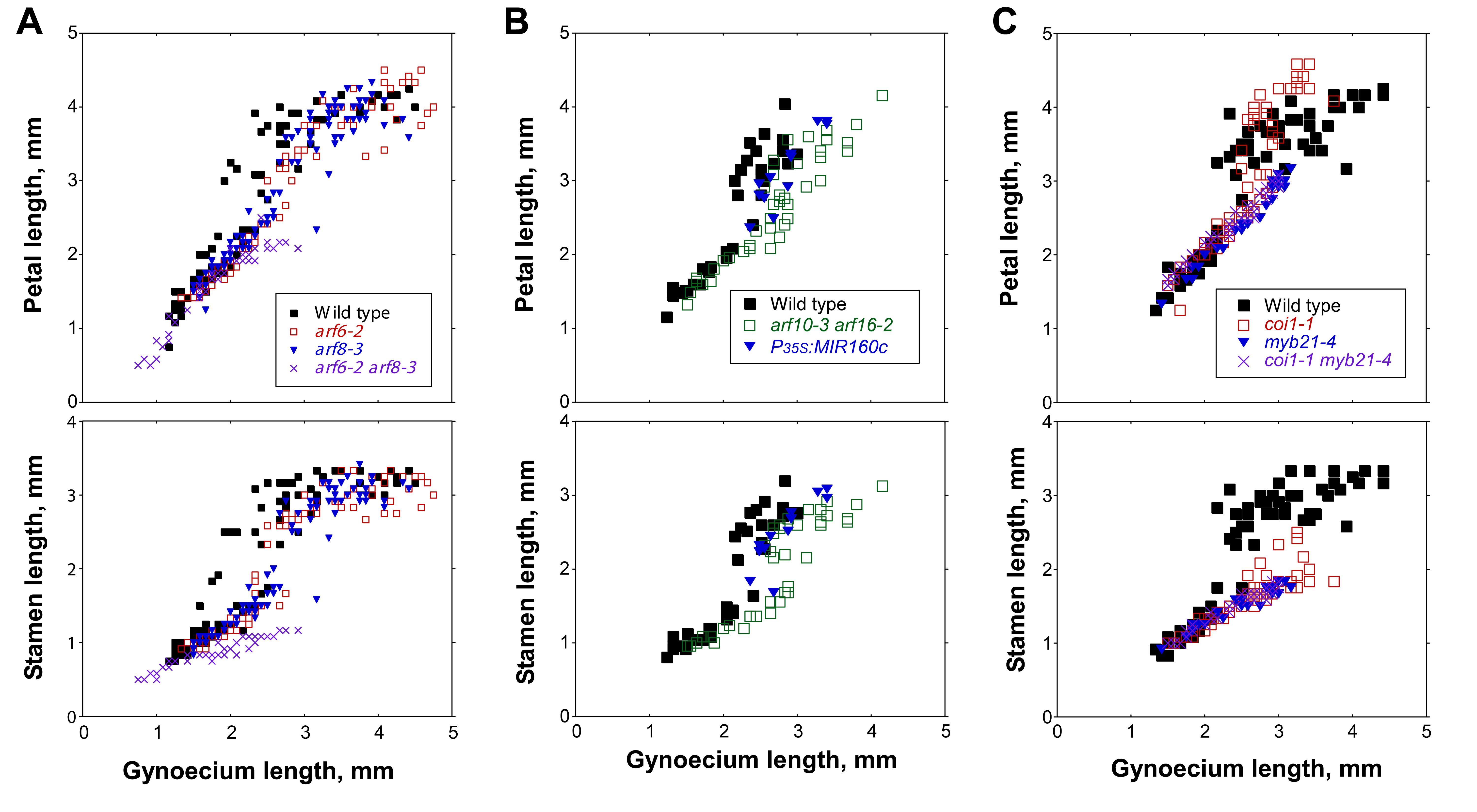

Supplement: Figure S2 — Petal and stamen lengths relative to gynoecium length for individual flowers. (A) Wild-type, arf6-2, arf8-3, and arf6-2 arf8-3 flowers. (B) Wild-type, arf10-3 arf16-2, and P35S:MIR160c flowers. The microRNA miR160 targets both ARF10 and ARF16. (C) Wild-type, coi1-1, myb21-4, and coi1-1 myb21-4 flowers. (TIF) [file pgen.1002506.s002.tif]

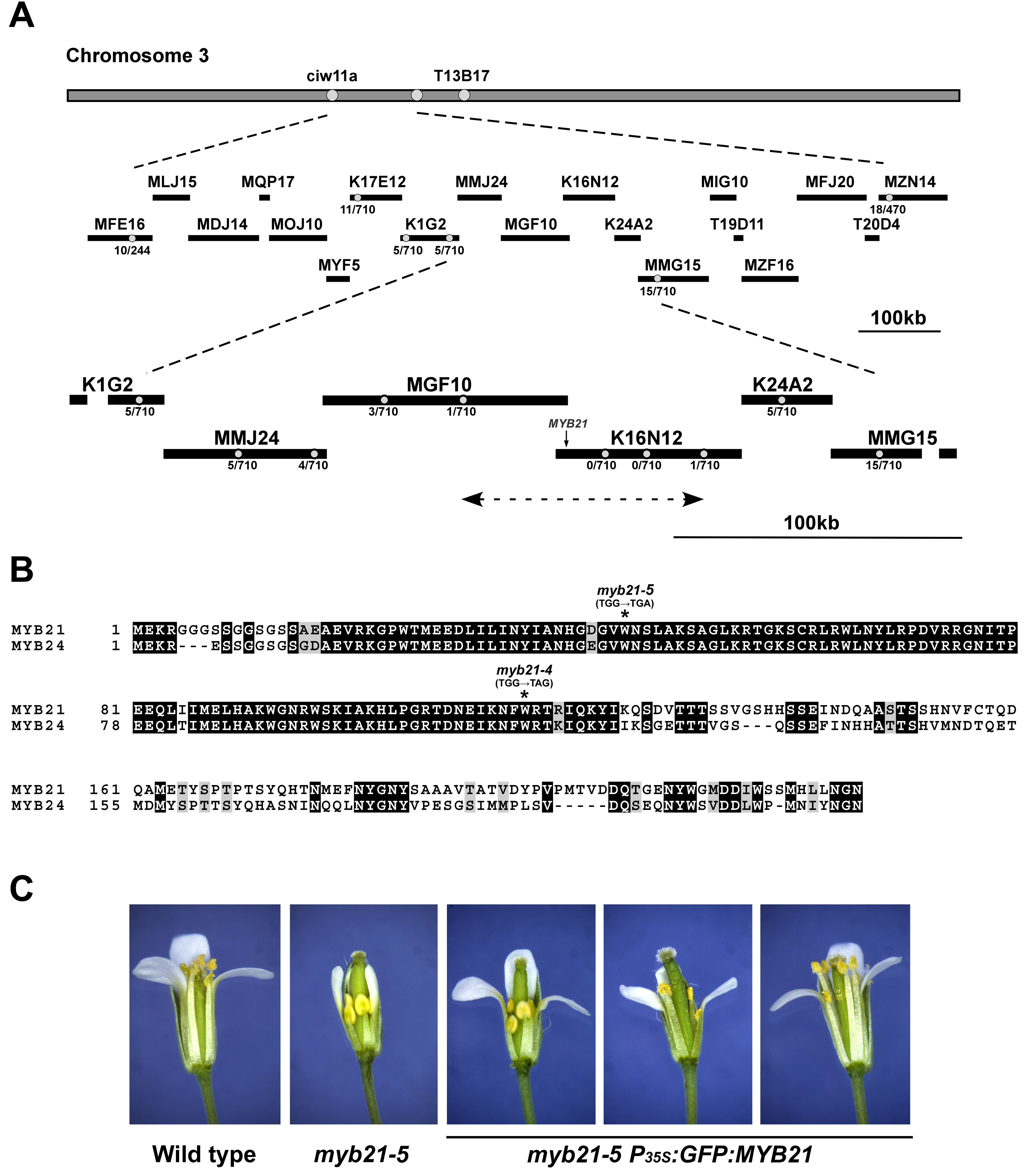

Supplement: Figure S3 — Map-based cloning of MYB21. (A) Map-based cloning of MYB21. Bulked segregant analysis was used to establish linkage of the arf6 enhancer (myb21-5) to marker ciw11a on chromosome 3. 355 arf6-2 myb21-like F2 plants from an arf6-2 myb21-5×arf6-2 (La-er) cross were then screened with PCR-based markers closely linked to ciw11a. One crossover event was detected between myb21-5 and each of the markers MGF10-40054 and K16N12-45751, indicating that the myb21-5 mutation was located within a 91 kb interval between these two markers. Sequencing of the MYB21 gene in this interval identified premature stop codons in both myb21-4 and myb21-5 alleles. (B) Alignment of predicted MYB21 and MYB24 amino acid sequences. Identical residues are shaded black, similar residues are shaded grey. Asterisks (*) indicate positions of the myb21-4 and myb21-5 point mutations. (C) Photographs of stage 13 flowers of wild type, myb21-5 and three independent myb21-5 P35S:GFP:MYB21 lines. The P35S:GFP:MYB21 transgene can restore petal and stamen elongation to the myb21-5 plant, and rescue the anther dehiscence defect. The flowers shown are the first open flower on wild-type inflorescence (stage 13, flower position 1), or its equivalent based upon bud size and position on the inflorescence stem. (TIF) [file pgen.1002506.s003.tif]

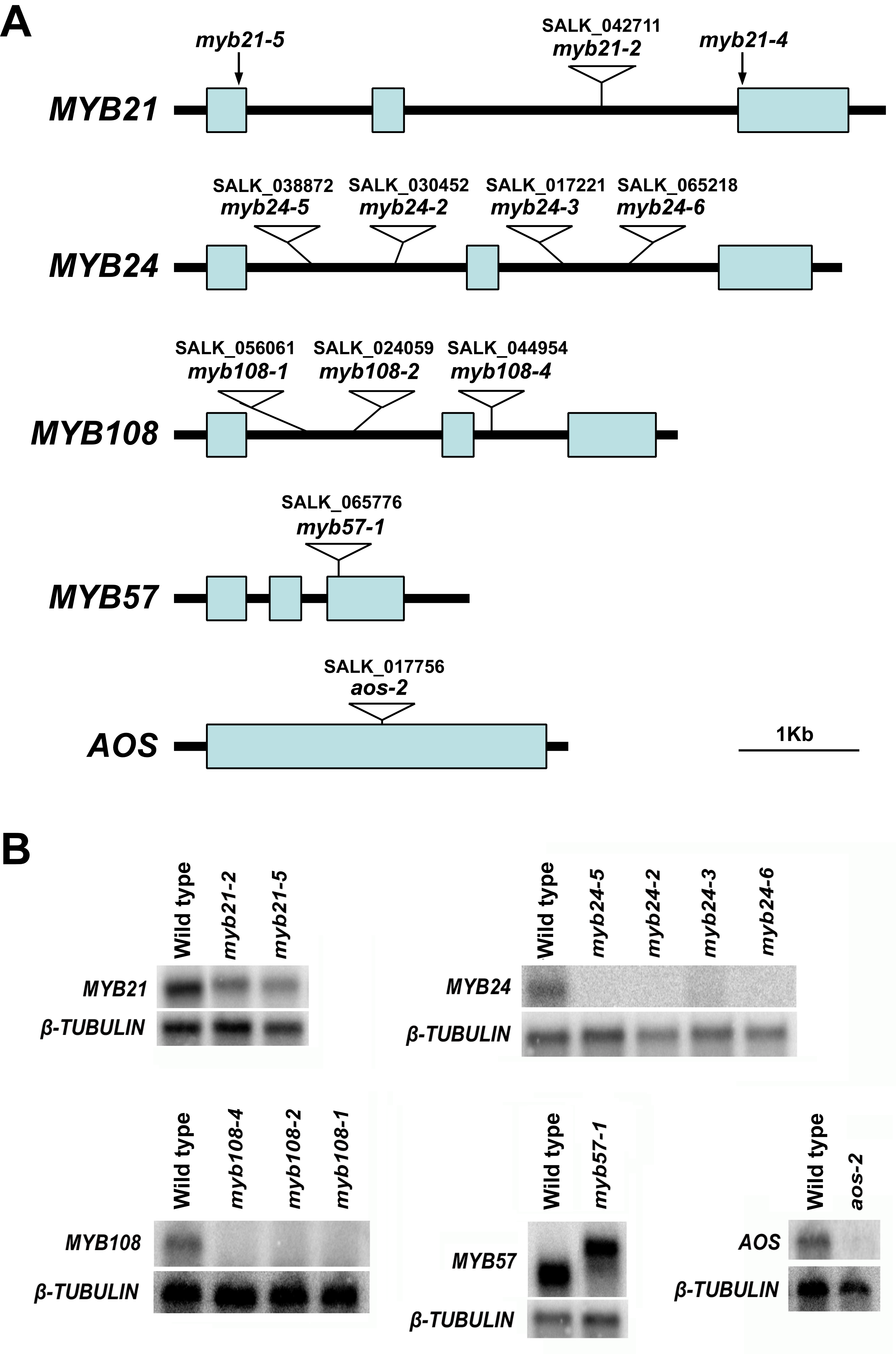

Supplement: Figure S4 — Mutations in MYB21, MYB24, MYB108, MYB57 and AOS. (A) Positions of mutations in MYB21, MYB24, MYB108, MYB57 and AOS genes. Exons are shown as blue boxes, T-DNA insertions are shown as triangles. The positions of T-DNA insertions are based upon sequencing provided by SIGNAL database [92]. (B) RNA gel blot hybridizations using MYB21, MYB24, MYB108, MYB57 and AOS probes. RNA was isolated from flowers of wild-type and homozygous T-DNA insertion mutant plants. A β-tubulin probe was used as a loading control. The transcript in myb21-2 flowers was confirmed to be MYB21 by sequencing RT-PCR products from the mutant. In some blots, the myb24-5 mutant had a transcript of a larger size, possibly arising from fusion to T-DNA sequences, that is not shown in the figure. (TIF) [file pgen.1002506.s004.tif]

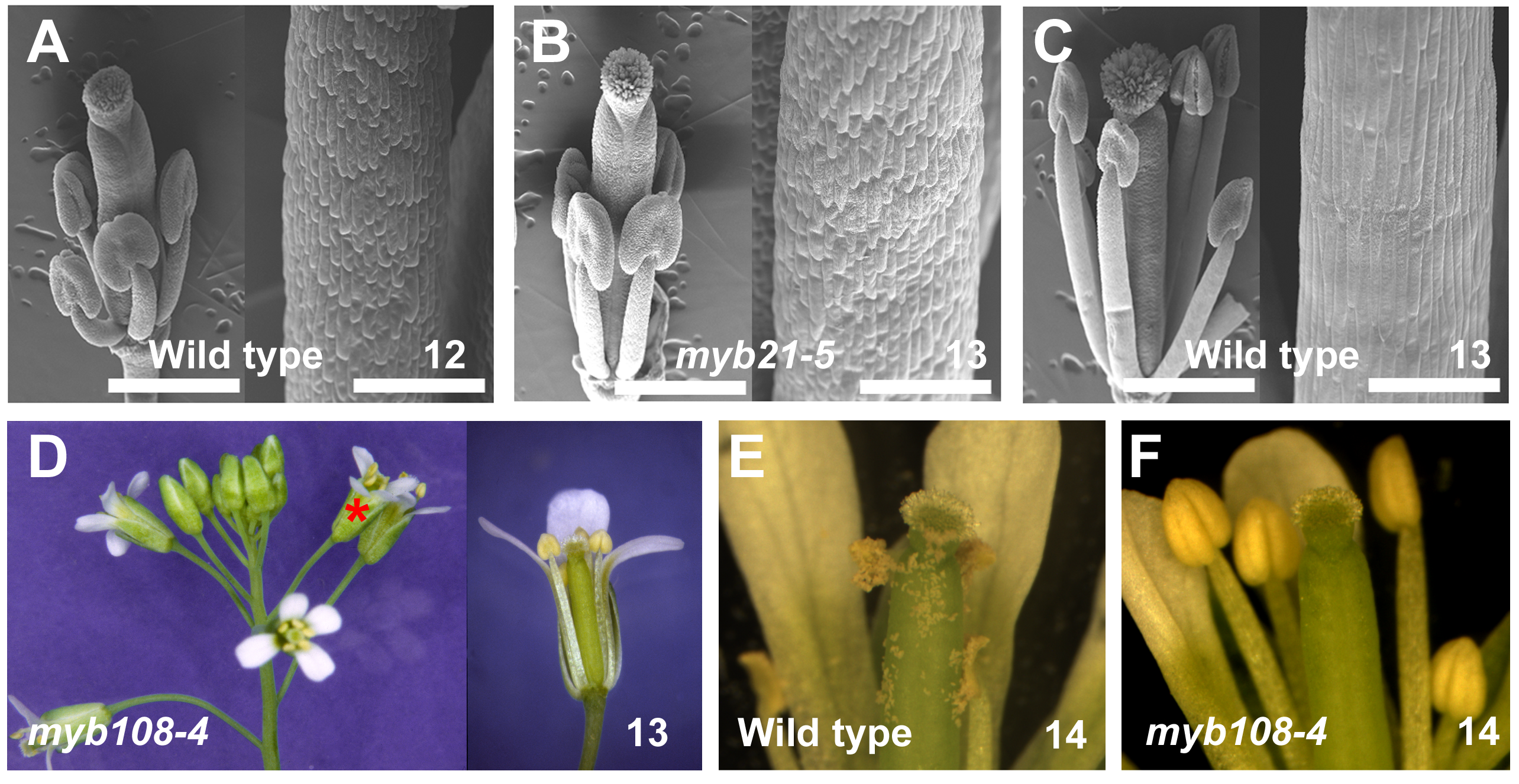

Supplement: Figure S5 — myb21-5 and myb108-4 flower phenotypes. (A–C) Scanning electron micrographs of stage 12 and 13 wild-type flowers (A,C) and stage 13 myb21-5 flower (B). For each picture, the left panel shows a flower with intact gynoecium and stamens (and perianth organs removed), and the right panel shows a closeup of the stamen filament. Scale bars: 800 µm (left panels); 100 µM (right panels). (D) Photographs of myb108-4 inflorescence and stage 13 flower. Asterisk in left panel indicates first open flower. (E,F) Closeup photographs of stage 14 wild-type and myb108-4 flowers, showing failure of anther dehiscence in the mutant. (TIF) [file pgen.1002506.s005.tif]

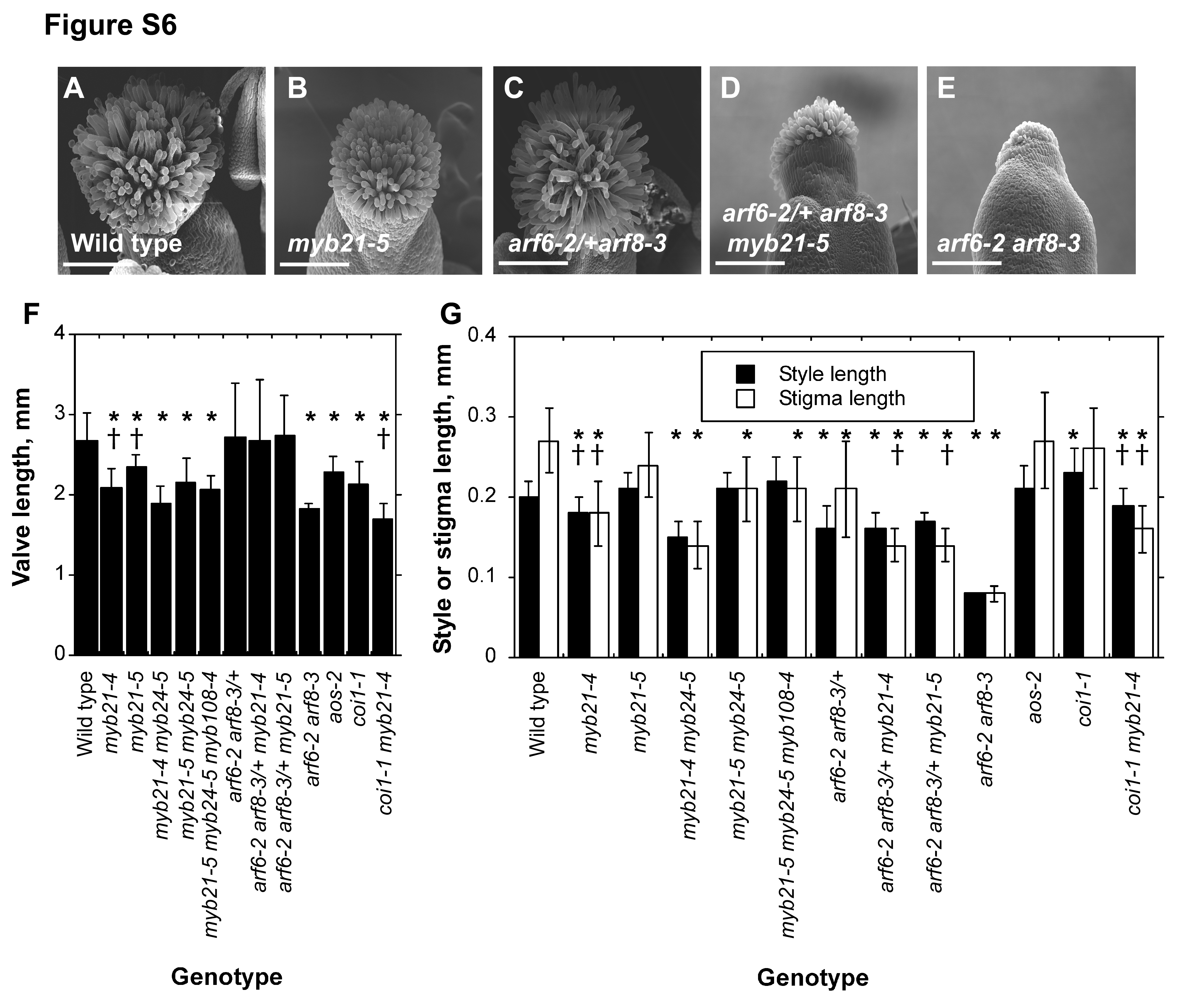

Supplement: Figure S6 — Gynoecium phenotypes of myb21 and arf6 arf8 mutants. (A–E) Scanning electron micrographs of stigmas from (A) wild-type (stage 13), (B) myb21-5 (stage 13), (C) arf6-2/ARF6 arf8-3 (stage 14), (D) arf6-2/ARF6 arf8-3 myb21-5 (stage 14), and (E) arf6-2 arf8-3 (stage 14) flowers. Scale bar = 200 µm. (F) Lengths of valves of stage 13–14 flowers of indicated genotypes. (G) Lengths of styles and stigmas of stage 13–14 flowers of indicated genotypes. Values in F and G are means ± SD for between 8 and 50 measurements. * indicates difference from corresponding wild-type measurement by t-test with P<0.001; † indicates significant difference from value for corresponding MYB21+ genotype by t-test with P<0.001. In A, F and G, wild-type flowers were emasculated 2-3 days before measurements, to prevent pollination and subsequent stigma collapse. (TIF) [file pgen.1002506.s006.tif]

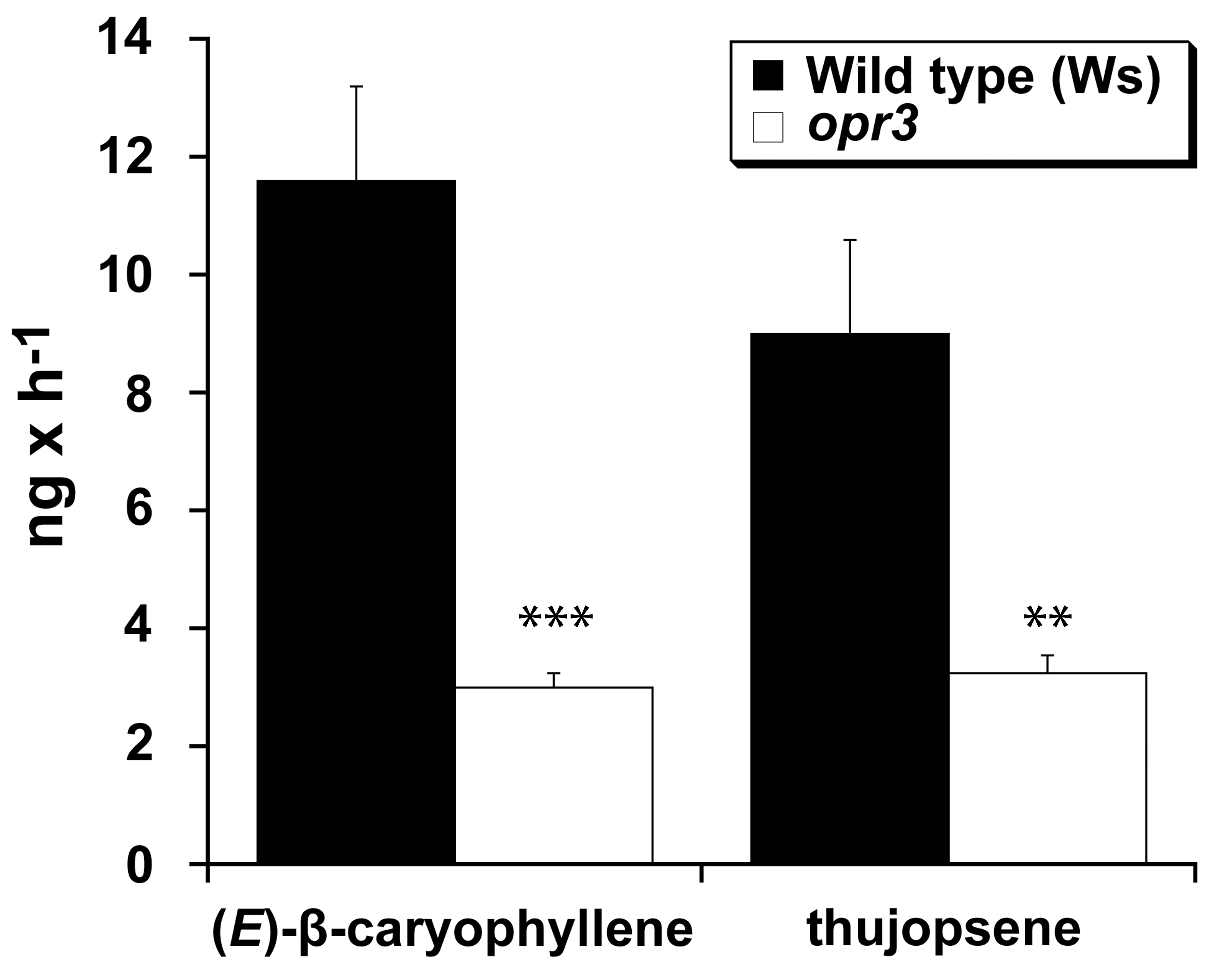

Supplement: Figure S7 — Volatile sesquiterpene emissions from wild-type and opr3 inflorescences. Emissions of (E)-β-caryophyllene and thujopsene are shown. Volatile compounds were collected from 70 inflorescences for 9 h by a closed-loop stripping procedure. The wild-type and opr3 plants were in the Wassilewskija (Ws) ecotype. Student's t-test,***, p<0.001; **, p<0.01. (TIF) [file pgen.1002506.s007.tif]
